# Sweet Battle of the Epimers—Continued Exploration of Monosaccharide-Derived Delivery Agents for Boron Neutron Capture Therapy

**DOI:** 10.1021/acs.molpharmaceut.3c00119

**Published:** 2023-05-03

**Authors:** Jelena Matović, Katayun Bahrami, Philipp Stockmann, Iris K. Sokka, You Cheng Khng, Mirkka Sarparanta, Evamarie Hey-Hawkins, Jarkko Rautio, Filip S. Ekholm

**Affiliations:** † Department of Chemistry, University of Helsinki, Finland, P.O. Box 55, Helsinki FI-00014, Finland; ‡ School of Pharmacy, University of Eastern Finland, P.O. Box 1627, Kuopio FI-70211, Finland; § Faculty of Chemistry and Mineralogy, Institute of Inorganic Chemistry, Leipzig University, Leipzig D-04103, Germany

**Keywords:** boron neutron capture therapy, carboranes, carbohydrates, drug delivery, medicinal chemistry

## Abstract

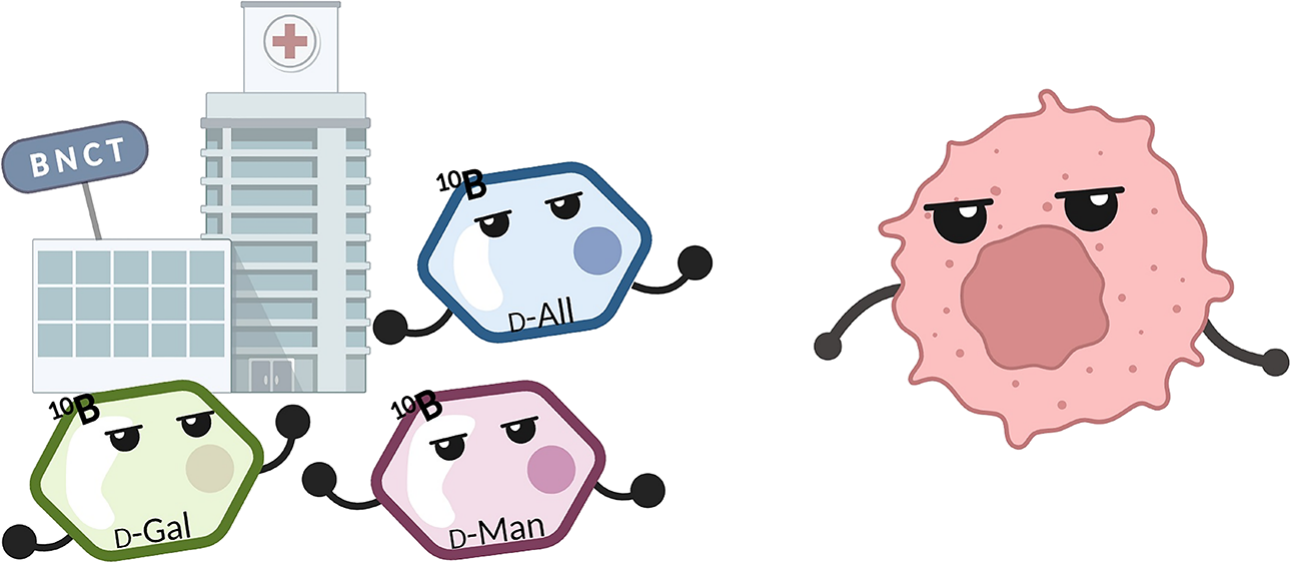

Boron neutron capture therapy (BNCT) is a cancer therapy in which boron delivery agents play a crucial role. In theory, delivery agents with high tumor targeting capabilities can lead to selective eradication of tumor cells without causing harmful side effects. We have been working on a GLUT1-targeting strategy to BNCT for a number of years and found multiple promising hit compounds which outperform the clinically employed boron delivery agents in vitro. Herein, we continue our work in the field by further diversification of the carbohydrate scaffold in order to map the optimal stereochemistry of the carbohydrate core. In the sweet battle of the epimers, carborane-bearing d-galactose, d-mannose, and d-allose are synthesized and subjected to in vitro profiling studies—with earlier work on d-glucose serving as the reference. We find that all of the monosaccharide delivery agents display a significantly improved boron delivery capacity over the delivery agents approved for clinical use in vitro, thus providing a sound foundation for advancing toward in vivo preclinical assessment studies.

## 1 Introduction

**Figure 1:**
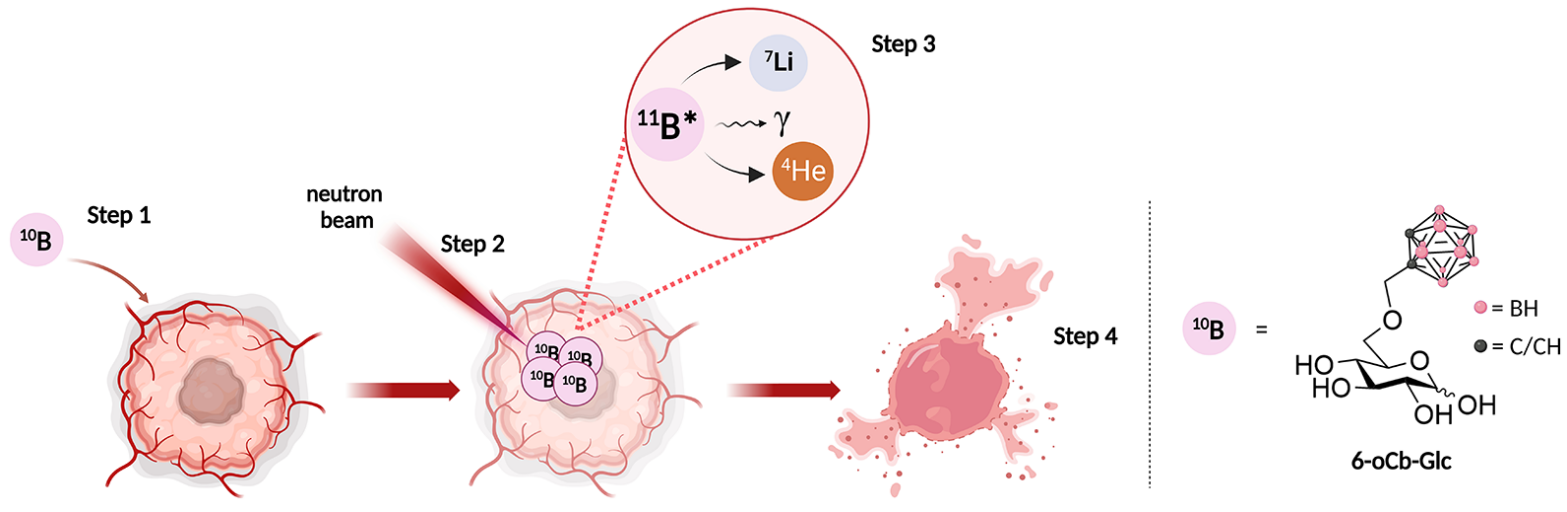
Left: graphical representation of the BNCT working principle. Step 1: ^10^B atoms are delivered selectively to cancer cells. Step 2: cancer cells are irradiated with a beam of low energy thermal neutrons. Step 3: upon irradiation, ^10^B undergoes a nuclear capture followed by a fission reaction, giving rise to ^7^Li and ^4^He nuclei (α particles). Step 4: ^4^He causes a destructive effect on the cancer cell. Right: an example of a promising GLUT1-targeting delivery agent from our recent work.^16^ Figure created with BioRender (https://biorender.com/).

Over the past 70 years, boron neutron capture therapy (BNCT) has steadily been developing as a promising cancer therapy.^1,2^ More recently, the installation of accelerator-based neutron sources in hospital environments has changed the state-of-the-art treatment possibilities simultaneously fueling a renewed interest in the potential of BNCT.^3^ While so, it is important to understand that the overall treatment prospects of BNCT will always be heavily tied to the performance of delivery agents and delivery strategies. In BNCT, the goal is to deliver a sufficient amount of ^10^B atoms selectively to tumor cells and irradiate the tumor region with thermal neutrons (Figure 1).^4−6^ The irradiation leads to a nuclear capture and fission reaction with a destructive magnitude that approximately equals the size of a single cell (approximately 5–9 μm). Thus, a highly selective tumor-targeting agent provides the best foundation for reaching a high ^10^B-distribution gradient between tumor and healthy tissue, a pre-requisite for a successful treatment outcome. Previous medicinal chemistry campaigns, aimed at improved delivery agents, have indicated the challenges involved and supplied a substantial list of criteria that ideal ^10^B-delivery agents need to fulfill in order to become suitable for BNCT.^5,7−11^ The most important ones relate to selectivity, i.e., high tumor-to-blood (T/B) and tumor-to-normal tissue ratios (T/N) (minimum of 3:1, ideally closer to 10:1), low systemic toxicity (otherwise other emerging cancer therapies may be better), boron delivery capacity in the range of 20−35 μg/1 g of tumor tissue,^5^ reasonable production costs, well-proven mechanism of action, and known metabolic fate. Despite the significant efforts invested in the development of improved delivery agents by a large number of research teams,^5,7−11^ only three delivery agents [boronophenylalanine (BPA),^12^ sodium borocaptate (BSH),^13^ and sodium decaborate (GB-10)^14,15^] have been approved for clinical use with none displaying optimal properties. Therefore, there is a need for new and improved delivery agents.

Since the initial work by Hawthorne and co-workers in 1988 on glycoconjugate delivery agents for BNCT,^17^ many attempts have been made to capitalize on their significant potential.^10,18,19^ Our team has in recent years contributed to the exploration of a GLUT1-targeting strategy to BNCT.^16,20,21^ The GLUT1-targeting approach is appealing as it is already a clinically validated tumor-targeting strategy applied in the diagnosis and staging of many types of cancers [most prominent example is the use of 2-deoxy-2-[fluorine-18]fluoro-d-glucose (^18^F-FDG) in positron emission tomography (PET) imaging].^22−24^ Adequate tumor-to-healthy tissue selectivity can in theory be reached through the “Warburg effect”,^25^ which describes the inefficient d-glucose metabolism in cancer cells leading to an overexpression of GLUT1 transporters on the cell membrane and an increased d-glucose uptake. Our previous work has showcased that carefully designed d-glucose-based delivery agents for BNCT are able to outperform the delivery agents in current clinical use in vitro.^16,20,21^ In fact, our prime candidate (Figure 1, right, **6-oCb-Glc**) was found to have a 40-fold boron delivery capacity compared to BPA and BSH.^16^ Nevertheless, the synthesis and in vitro assessment of positional isomers and different connecting atoms in the carborane cluster revealed that structurally similar glucose-based delivery agents may not be equally promising.^20,21^

**Figure 2:**
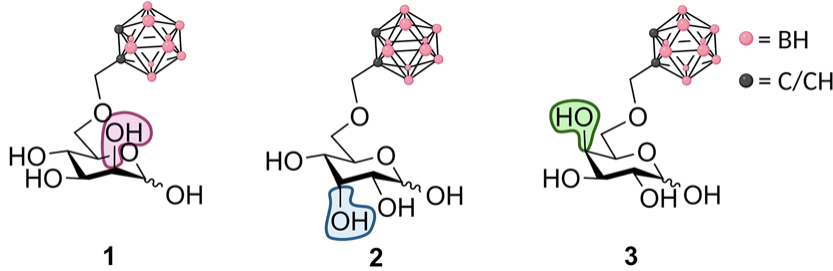
Molecular structures of the three synthesized and studied glycoconjugates. **1** is based on d-Man, **2** on d-All, and **3** on d-Gal.

In our continued work to map the boundaries of the GLUT1-targeting approach and optimize the delivery agents, we herein focus on assessment of the stereochemistry in the carbohydrate core. This is a valid approach as the GLUT family is known to accept a variety of monosaccharides.^26,27^ In addition, these monosaccharides may display altered uptake profiles through other potential hexose transporters, thus affecting their boron delivery capacity. Therefore, we set out to synthesize and assess the potential of carboranylmethyl-bearing epimers of d-glucose (Glc), i.e., d-mannose (Man), d-galactose (Gal), and d-allose (All) (see Figure 2). We limited the epimer set to species in which the carborane cluster is installed at the position 6 as this was found to be the most promising delivery agent in the d-Glc series.^20^ The assessment of their potential (GLUT1 affinity, toxicity, and boron delivery capacity) was performed in the CAL 27 oral adenosquamous carcinoma cell line^23,28^ in order to be able to compare the results to those obtained earlier in the Glc series.^16,20^

## 2 Experimental Section

### 2.1 Synthesis and Structural Characterization

All reaction protocols requiring inert reaction conditions were performed under an argon atmosphere using dry solvents (purified with VAC vacuum solvent purification system and additionally dried over molecular sieves). Reagents and commercially available starting material were purchased from commercial sources. All NMR spectra were recorded with a 500 MHz Bruker Avance III NMR spectrometer (^1^H: 499.83 MHz, ^13^C: 125.69 MHz, ^11^B: 160.36 MHz). All products were characterized with 1D: ^1^H, ^13^C{^1^H}, ^11^B{^1^H}, and 1D-TOCSY and 2D-techniques: ed-HSQC, HMBC, and COSY by using pulse sequences provided by the instrument’s manufacturer. Chemical shifts are expressed on the δ scale (in ppm) using TMS, residual chloroform, methanol, or 15% BF_3_ in CDCl_3_ (^11^B NMR) as internal standards. Coupling constants were obtained through spectral simulations with the ChemAdder spectral simulation software. They are given in Hz and provided only once, when first encountered. Coupling patterns are given as s (singlet), d (doublet), dd (doublet of a doublet), etc. HRMS spectra were recorded using a Bruker Micro Q-TOF with ESI (electrospray ionization) operated in positive mode. The purity of glycoconjugates **1–3** was determined by qNMR studies using maleic acid as an internal standard. TLC was performed on aluminum sheets precoated with silica gel 60F254 (Merck), and spots were visualized by spraying with conc. H_2_SO_4_:MeOH (1:5) and heating. Flash chromatography was carried out on silica gel 40.

#### 2.1.1 General Experimental Procedures

##### 2.1.1.1 General Procedure for Silylation of the Primary 6-OH Group in a Monosaccharide

The corresponding monosaccharide (1.0 equiv) was dissolved in dry pyridine (10 mL/g of starting material) and brought to 0 °C using an ice bath. *tert*-Butyldimethylsilyl chloride (1.1 equiv) was slowly added to the reaction mixture. After the addition of the silylating agent, the reaction mixture was brought to r.t. and stirred for 2–4 h. Upon completion of the reaction, the solvent was removed under reduced pressure, and the crude product was purified by column chromatography (DCM:MeOH 8:1).

##### 2.1.1.2 General Procedure for Alkylation of a Free OH Group

The corresponding carbohydrate was dissolved in dry DMF (2 mL/100 mg of starting material) and brought to 0 °C using an ice bath. NaH (60% in mineral oil, 1.6 equiv/OH group) was added slowly to the reaction mixture and left to stir for 15 min. The reaction mixture was brought to r.t., and the corresponding alkyl bromide (1.5 equiv/OH group) was added. The mixture was left to stir for 2–3 h. After the reaction was completed, the reaction mixture was quenched by the addition of MeOH (0.4 mL/mmol of starting material), diluted with DCM (4 mL/100 mg of starting material), and washed with a saturated aqueous solution of NaHCO_3_ (3 mL/100 mg of starting material). The aqueous phase was extracted with DCM (3 times 3 mL/100 mg of starting material). The combined organic phases were washed with brine (3 mL/100 mg of starting material), dried over anhydrous MgSO_4_, filtered, and concentrated. The crude product was purified by column chromatography (EtOAc:Hex 1:3).

##### 2.1.1.3 General Procedure for Deprotection of the Silyl Group Using HF-Pyridine

The silylated glycoside (1.0 equiv) was dissolved in dry THF (3 mL/0.2 g of starting material) at 0 °C, and HF·pyridine (18 μL/0.03 mmol of starting material) was added to the reaction mixture. The resulting mixture was slowly brought to r.t. and stirred for 18 h. The reaction mixture was diluted with DCM (30 mL/0.5 g of starting material) and quenched with the addition of saturated aqueous solution of NaHCO_3_ (20 mL/0.2 g of starting material). The aqueous phase was extracted with DCM (3 times 20 mL/0.5 g of starting material). Combined organic phases were washed with brine (20 mL/0.5 g of starting material). The combined organic phases were dried over anhydrous MgSO_4_, filtered, and concentrated. The crude product was purified by column chromatography (EtOAc:Hex 1:2).

##### 2.1.1.4 General Procedure for Installation of the Carboranyl Moiety

B_10_H_14_ (1.6 equiv) was dissolved in dry MeCN (5 mL/150 mg of starting material) and heated to 60 °C. After 1 h, the propargylated glycoside (1.0 equiv) was dissolved in dry toluene (5 mL/150 mg of starting material) and added to the reaction mixture. Upon the addition, the temperature was increased to 80 °C, and the reaction mixture was left to stir for 16 h. Upon reaction completion, the reaction mixture was quenched with dry MeOH (1.5 mL/150 mg of starting material) and stirred for an additional 30 min at 80 °C. The solvents were removed under reduced pressure, and the crude product was purified by column chromatography (EtOAc:Hex 1:3).

##### 2.1.1.5 General Procedure for Deprotection of Benzyl Groups

The benzyl-protected glycoside was dissolved in EtOAc:MeOH (7:1, 0.1 mL/100 mg of starting material). 10% Pd/C (1 weight equiv) was added to the reaction mixture, and the reaction vessel was placed in an autoclave. The reactor was flushed with N_2_ three times and then filled with H_2_ (4 bar). The reaction was left to stir for 4–6 h. Upon reaction completion, the resulting mixture was filtered through celite, washed with EtOAc:MeOH (7:1, 3 times 10 mL), and concentrated under reduced pressure. The crude product was purified by column chromatography (DCM:MeOH 7:1).

##### 2.1.1.6 General Procedure for Removal of Acetal Protecting Groups

The corresponding protected glycoside (1.0 equiv) was dissolved in Et_2_O. TFA and H_2_O were added to the reaction mixture (TFA/H_2_O/Et_2_O, 2:1:2, 10 mL/0.2 g of starting material) and left to stir at r.t. for 24 h. The resulting mixture was concentrated under reduced pressure, and the crude product was purified by column chromatography (DCM:MeOH 7:1).

#### 2.1.2 Substrate-Specific Analytical Data

##### 2.1.2.1 6-*O*-(*tert*-Butyldimethylsilyl)-α-d-mannopyranose

This compound was synthesized from d-mannose (2.35 g, 13.0 mmol) and TBDMSCl (2.15 g, 14.3 mmol) according to the general procedure for silylation of the primary 6-OH group of monosaccharides. This reaction gave the title compound as a white solid (2.60 g, 68%). TLC: *R*_f_: 0.61 (DCM:MeOH 5:1).

^1^H NMR (499.83 MHz, MeOD, 25 °C): δ = 5.05 (d, 1H, *J*_1,2_ = 1.8 Hz, H-1), 3.94 (dd, 1H, *J*_5,6a_ = 2.4, *J*_6a,6b_ = −11.4 Hz, H-6a), 3.83 (dd, 1H, *J*_5,6b_ = 5.5 Hz, H-6b), 3.77 (dd, 1H, *J*_2,3_ = 3.4 Hz, H-2), 3.75 (dd, 1H, *J*_3,4_ = 9.2 Hz, H-3), 3.74 (ddd, 1H, *J*_4,5_ = 9.2 Hz, H-5), 3.59 (dd, 1H, H-4), 0.92 (s, 9H, 6-OSi(CH_3_)_2_C(C**H_3_**)_3_) and 0.10 and 0.09 (each s, each 3H, 6-OSi(C**H_3_**)_2_C(CH_3_)_3_) ppm.

^13^C{^1^H} NMR (125.69 MHz, MeOD, 25 °C): δ = 95.7 (C-1), 74.3 (C-3), 72.8 (C-2), 72.4 (C-5), 69.0 (C-4), 64.9 (C-6), 26.5 (6-OSi(CH_3_)_2_C(**C**H_3_)_3_), 19.3 (6-OSi-(CH_3_)_2_**C**(CH_3_)_3_) and −5.10 (6-OSi(**C**H_3_)_2_C(CH_3_)_3_) ppm.

HRMS: *m*/*z* calcd for C_12_H_26_O_6_SiNa [M + Na]^+^ 317.1397; found 317.1302.

##### 2.1.2.2 1,2,3,4-Tetra-*O*-benzyl-6-*O*-(*tert*-butyldimethylsilyl)-d-mannopyranoside

This compound was synthesized from 6-*O*-(*tert*-butyldimethylsilyl)-β-d-mannopyranose (1.21 g, 4.1 mmol), NaH (0.67 g, 27.9 mmol), and BnBr (3.88 g, 22.7 mmol) according to the general procedure for alkylation of a free OH group. This reaction gave the title compound as a colorless oil (2.14 g, 80%, α:β 25:75). TLC: *R*_f_: 0.8 (EtOAc:Hex 1:2).

α anomer: ^1^H NMR (499.83 MHz, CDCl_3_, 25 °C): δ = 7.46–7.23 (m, 20H, arom. H), 4.92 and 4.63 (each d, each 1H, *J* = −10.6 Hz, 4-OC**H_2_**Ph), 4.91 (d, 1H, *J*_1,2_ = 1.8 Hz, H-1), 4.71 and 4.65 (each d, each 1H, *J* = −12.6 Hz, 2-OC**H_2_**Ph), 4.68 and 4.42 (each d, each 1H, *J* = −10.0 Hz, 1-OC**H_2_**Ph), 4.64 and 4.55 (each d, each 1H, *J* = −10.1 Hz, 3-OC**H_2_**Ph), 3.95 (dd, 1H, *J*_2,3_ = 3.2, *J*_3,4_ = 9.5 Hz, H-3), 3.94 (dd, 1H, *J*_4,5_ = 9.6 Hz, H-4), 3.86 (dd, 1H, *J*_5,6a_ = 1.0, *J*_6a,6b_ = −10.5 Hz, H-6a), 3.85 (dd, 1H, *J*_5,6b_ = 5.0 Hz, H-6b), 3.79 (dd 1H, H-2), 3.67 (ddd, 1H, H-5), 0.90 (s, 9H, 6-OSi(CH_3_)_2_C(C**H_3_**)_3_) and 0.09 and 0.07 (each s, each 3H, 6-OSi(C**H_3_**)_2_C(CH_3_)_3_) ppm.

^13^C{^1^H} NMR (125.69 MHz, CDCl_3_, 25 °C): δ = 139.0–127.2 (arom. C), 96.9 (C-1), 80.3 (C-3), 75.2 (C-2), 75.1 (4-O**C**H_2_Ph and 3-O**C**H_2_Ph), 75.0 (C-4), 73.5 (C-5), 72.5 (2-O**C**H_2_Ph), 68.6 (1-O**C**H_2_Ph), 62.8 (C-6), 26.0 (6-OSi(CH_3_)_2_C(**C**H_3_)_3_), 18.3 (6-OSi(CH_3_)_2_**C**(CH_3_)_3_) and −5.1 and −5.2 (6-OSi(**C**H_3_)_2_C(CH_3_)_3_ ppm.

β anomer: ^1^H NMR (499.83 MHz, CDCl_3_, 25 °C): δ = 7.46–7.23 (m, 20H, arom. H), 4.97 and 4.86 (each d, each 1H, *J* = −12.4 Hz, 2-OC**H_2_**Ph), 4.94 and 4.58 (each d, each 1H, *J* = −11.9 Hz, 1-OC**H_2_**Ph), 4.92 and 4.64 (each d, each 1H, *J* = −10.8 Hz, 4-OC**H_2_**Ph), 4.53 and 4.46 (each d, each 1H, *J* = −10.8 Hz, 4-OC**H_2_**Ph), 4.39 (d, 1H, *J*_1,2_ = 0.2 Hz, H-1), 3.92 (dd, 1H, *J*_5,6a_ = 1.7, *J*_6a,6b_ = −10.9 Hz, H-6a), 3.89 (dd, 1H, *J*_2,3_ = 3.1 Hz, H-2), 3.87 (dd, 1H, *J*_3,4_ = 9.5, *J*_4,5_ = 9.6 Hz, H-4), 3.86 (dd, 1H, *J*_5,6b_ = 5.6 Hz, H-6b), 3.47 (dd 1H, H-3), 3.26 (ddd, 1H, H-5), 0.90 (s, 9H, 6-OSi(CH_3_)_2_C(C**H_3_**)_3_) and 0.09 and 0.07 (each s, each 3H, 6-OSi(C**H_3_**)_2_C(CH_3_)_3_) ppm.

^13^C{^1^H} NMR (125.69 MHz, CDCl_3_, 25 °C): δ = 139.0–127.2 (arom. C), 99.9 (C-1), 82.3 (C-3), 77.1 (C-5), 75.1 (4-O**C**H_2_Ph), 74.9 (C-4), 74.1 (C-2), 73.7 (2-O**C**H_2_Ph), 71.4 (3-O**C**H_2_Ph), 70.4 (1-O**C**H_2_Ph), 62.9 (C-6), 25.9 (6-OSi(CH_3_)_2_C(**C**H_3_)_3_), 18.4 (6-OSi(CH_3_)_2_**C**(CH_3_)_3_) and −5.1 and −5.2 (6-OSi(**C**H_3_)_2_C(CH_3_)_3_ ppm.

HRMS: *m*/*z* calcd for C_40_H_50_O_6_SiNa [M + Na]^+^ 677.3275; found 677.3036.

##### 2.1.2.3 1,2,3,4-Tetra-*O*-benzyl-d-mannopyranoside (**4**)

This compound was synthesized from 1,2,3,4-tetra-*O*-benzyl-6-*O*-(*tert*-butyldimethylsilyl)-d-mannopyranoside (2.04 g, 3.12 mmol) according to the general procedure for silyl group removal using HF·pyridine. This reaction gave the title compound as a white solid (1.01 g, 60%, α:β 36:64). TLC: *R*_f_: 0.34 (EtOAc:Hex 1:2).

α anomer: ^1^H NMR (499.83 MHz, CDCl_3_, 25 °C): δ = 7.46–7.22 (m, 20H, arom. H), 4.94 and 4.62 (each d, each 1H, *J* = −10.9 Hz, 4-OC**H_2_**Ph), 4.90 (d, 1H, *J*_1,2_ = 1.8 Hz, H-1), 4.88 and 4.64 (each d, each 1H, *J* = −10.1 Hz, 3-OC**H_2_**Ph), 4.75 and 4.66 (each d, each 1H, *J* = −12.5 Hz, 2-OC**H_2_**Ph), 4.65 and 4.43 (each d, each 1H, *J* = −11.4 Hz, 1-OC**H_2_**Ph), 4.00 (dd, 1H, *J*_3,4_ = 9.2, *J*_4,5_ = 8.8 Hz, H-4), 3.97 (dd, 1H, *J*_2,3_ = 2.6 Hz, H-3), 3.92 (ddd, 1H, *J*_5,6a_ = 3.1, *J*_6a,6b_ = −10.1, *J*_6a,6-OH_ = 3.8 Hz, H-6a), 3.82 (dd 1H, H-2), 3.78 (ddd, 1H, *J*_5,6b_ = 5.1, *J*_6b,6-OH_ = 6.4 Hz, H-6b), 3.70 (ddd, 1H, H-5) and 2.02 (dd, 1H, 6-OH) ppm.

^13^C{^1^H} NMR (125.69 MHz, CDCl_3_, 25 °C): δ = 138.7–127.6 (arom. C), 97.7 (C-1), 80.3 (C-3), 75.4 (4-O**C**H_2_Ph), 75.0 (C-4), 74.9 (C-2), 73.0 (2-O**C**H_2_Ph), 72.5 (C-5), 72.4 (3-O**C**H_2_Ph), 69.2 (1-O**C**H_2_Ph) and 62.5 (C-6) ppm.

β anomer: ^1^H NMR (499.83 MHz, CDCl_3_, 25 °C): δ = 7.46–7.22 (m, 20H, arom. H), 4.98 and 4.88 (each d, each 1H, *J* = −12.4 Hz, 2-OC**H_2_**Ph), 4.95 and 4.60 (each d, each 1H, *J* = −12.0 Hz, 1-OC**H_2_**Ph), 4.94 and 4.64 (each d, each 1H, *J* = −10.9 Hz, 4-OC**H_2_**Ph), 4.52 and 4.47 (each d, each 1H, *J* = −11.8 Hz, 3-OC**H_2_**Ph), 4.48 (d, 1H, *J*_1,2_ = 0.8 Hz, H-1), 3.94 (dd, 1H, *J*_3,4_ = 9.3, *J*_4,5_ = 9.4 Hz, H-4), 3.93 (dd, 1H, *J*_2,3_ = 2.9 Hz, H-2), 3.91 (ddd, 1H, *J*_5,6a_ = 2.6, *J*_6a,6b_ = −12.8, *J*_6a,6-OH_ = 4.7 Hz, H-6a), 3.78 (ddd, 1H, *J*_5,6b_ = 5.9, *J*_6b,6-OH_ = 6.6 Hz, H-6b), 3.51 (dd, 1H, H-3), 3.32 (ddd, 1H, H-5) and 2.15 (dd, 1H, 6-OH) ppm.

^13^C{^1^H} NMR (125.69 MHz, CDCl_3_, 25 °C): δ = 138.7–127.6 (arom. C), 100.6 (C-1), 82.5 (C-3), 76.0 (C-5), 75.4 (4-O**C**H_2_Ph), 75.0 (C-4), 74.2 (C-2 and 2-O**C**H_2_Ph), 71.7 (3-O**C**H_2_Ph), 71.3 (1-O**C**H_2_Ph) and 62.7 (C-6) ppm.

HRMS: *m*/*z* calcd for C_34_H_36_O_6_Na [M + Na]^+^ 563.2410; found 563.3879.

##### 2.1.2.4 1,2,3,4-Tetra-*O*-benzyl-6-*O*-propargyl-d-mannopyranoside (**5**)

This compound was synthesized from **4** (0.94 g, 1.7 mmol), NaH (0.07 g, 3.0 mmol), and propargyl bromide (0.21 g, 1.8 mmol) according to the general procedure for alkylation of a free OH group. This reaction gave the title compound as a colorless oil (0.69 g, 70%, α:β 29:71). TLC: *R*_f_: 0.71 (EtOAc:Hex 1:2).

α anomer: ^1^H NMR (499.83 MHz, CDCl_3_, 25 °C): δ = 7.46–7.22 (m, 20H, arom. H), 4.94 (d, 1H, *J*_1,2_ = 1.8 Hz, H-1), 4.92 and 4.67 (each d, each 1H, *J* = −10.7 Hz, 4-OC**H_2_**Ph), 4.71 and 4.70 (each d, each 1H, *J* = −13.2 Hz, 2-OC**H_2_**Ph), 4.69 and 4.44 (each d, each 1H, *J* = −12.0 Hz, 1-OC**H_2_**Ph), 4.62 and 4.60 (each d, each 1H, *J* = −11.7 Hz, 3-OC**H_2_**Ph), 4.28 (dd, 1H, *J*_CH,CH2a_ = −2.4, *J*_CH2a,CH2b_ = −15.9 Hz, 6-OC**H_2a_**C≡CH), 4.21 (dd, 1H, *J*_CH,CH2b_ = −2.4 Hz, 6-OC**H_2b_**C≡CH), 4.01 (dd, 1H, *J*_3,4_ = 9.5, *J*_4,5_ = 9.8 Hz, H-4), 3.94 (dd, 1H, *J*_2,3_ = 3.2 Hz, H-3), 3.89 (dd, 1H, *J*_5,6a_ = 4.8, *J*_6a,6b_ = −10.5 Hz, H-6a), 3.82 (ddd, 1H, *J*_5,6b_ = 5.1 Hz, H-5), 3.80 (dd 1H, H-2), 3.75 (dd, 1H, H-6b) and 2.38 (dd, 1H, 6-OCH_2_C≡C**H**) ppm.

^13^C{^1^H} NMR (125.69 MHz, CDCl_3_, 25 °C): δ = 138.9–127.5 (arom. C), 97.5 (C-1), 80.3 (6-OCH_2_**C**≡CH), 80.0 (C-3), 75.3 (4-O**C**H_2_Ph), 74.9 (C-4), 74.7 (C-2), 74.6 (6-OCH_2_C≡**C**H), 72.7 (2-O**C**H_2_Ph), 72.3 (3-O**C**H_2_Ph), 71.9 (C-5), 69.2 (1-O**C**H_2_Ph), 68.9 (C-6) and 58.7 (6-O**C**H_2_C≡CH) ppm.

β anomer: ^1^H NMR (499.83 MHz, CDCl_3_, 25 °C): δ = 7.46–7.24 (m, 20H, arom. H), 5.00 and 4.88 (each d, each 1H, *J* = −12.5 Hz, 2-OC**H_2_**Ph), 4.99 and 4.58 (each d, each 1H, *J* = −12.0 Hz, 1-OC**H_2_**Ph), 4.94 and 4.67 (each d, each 1H, *J* = −11.8 Hz, 4-OC**H_2_**Ph), 4.50 and 4.44 (each d, each 1H, *J* = −12.0 Hz, 3-OC**H_2_**Ph), 4.42 (d, 1H, *J*_1,2_ = 0.7 Hz, H-1), 4.27 (dd, 1H, *J*_CH,CH2a_ = −2.4, *J*_CH2a,CH2b_ = −15.9 Hz, 6-OC**H_2a_**C≡CH), 4.24 (dd, 1H, *J*_CH,CH2b_ = −2.4 Hz, 6-OC**H_2b_**C≡CH), 3.93 (dd, 1H, *J*_3,4_ = 9.4, *J*_4,5_ = 9.7 Hz, H-4), 3.92 (dd, 1H, *J*_2,3_ = 3.0 Hz, H-2), 3.87 (dd, 1H, *J*_5,6a_ = 5.1, *J*_6a,6b_ = −12.2 Hz, H-6a), 3.86 (dd, 1H, *J*_5,6b_ = 2.3 Hz, H-6b), 3.48 (dd, 1H, H-3), 3.42 (ddd, 1H, H-5) and 2.38 (dd, 1H, 6-OCH_2_C≡C**H**) ppm.

^13^C{^1^H} NMR (125.69 MHz, CDCl_3_, 25 °C): δ = 138.9–127.5 (arom. C), 100.5 (C-1), 82.4 (C-3), 80.3 (6-OCH_2_**C**≡CH), 75.8 (C-5), 75.3 (4-O**C**H_2_Ph), 74.8 (C-4), 74.6 (6-OCH_2_C≡**C**H), 74.0 (2-O**C**H_2_Ph), 73.9 (C-2), 71.6 (3-O**C**H_2_Ph), 71.0 (1-O**C**H_2_Ph), 69.2 (C-6) and 58.8 (6-O**C**H_2_C≡CH) ppm.

HRMS: *m*/*z* calcd For C_37_H_38_O_6_Na [M + Na]^+^ 601.2566; found 601.2229.

##### 2.1.2.5 1,2,3,4-Tetra-*O*-benzyl-6-*O*-(*o*-carboranylmethyl)-β-d-mannopyranoside (**6**)

This compound was synthesized from **5** (0.60 g, 1.0 mmol) and B_10_H_14_ (0.20 g, 1.7 mmol) according to the general procedure for installation of the carboranyl moiety. This reaction gave the title compound as a white solid (0.37 g, 53%). TLC: *R*_f_: 0.70 (EtOAc:Hex 1:2).

^1^H NMR (499.83 MHz, CDCl_3_, 25 °C): δ = 7.44–7.23 (m, 20H, arom. H), 4.94 and 4.84 (each d, each 1H, *J* = −12.5 Hz, 4-OC**H_2_**Ph), 4.94 and 4.59 (each d, each 1H, *J* = −10.4 Hz, 2-OC**H_2_**Ph), 4.90 and 4.57 (each d, each 1H, *J* = −12.0 Hz, 1-OC**H_2_**Ph), 4.51 and 4.44 (each d, each 1H, *J* = −11.8 Hz, 3-OC**H_2_**Ph), 4.41 (d, 1H, *J*_1,2_ = 0.6 Hz, H-1), 4.06 (br s, 1H, carborane C**H**), 4.01 and 3.94 (each d, each 1H, *J* = −10.7 Hz, 6-OC**H_2_**-carborane), 3.91 (dd, 1H, *J*_2,3_ = 2.9 Hz, H-2), 3.85 (dd, 1H, *J*_3,4_ = 9.4, *J*_4,5_ = 9.7 Hz, H-4), 3.81 (dd, 1H, *J*_5,6a_ = 4.7, *J*_6a,6b_ = −11.6 Hz, H-6a), 3.64 (dd, 1H, *J*_5,6b_ = 1.8 Hz, H-6b), 3.46 (dd, 1H, H-3), 3.30 (ddd, 1H, H-5) and 2.98–1.36 (br m, 10H, carborane B**H**) ppm.

^13^C{^1^H} NMR (125.69 MHz, CDCl_3_, 25 °C): δ = 138.6–127.7 (arom. C), 100.5 (C-1), 82.3 (C-3), 75.8 (C-5), 75.3 (2-O**C**H_2_Ph), 74.2 (4-O**C**H_2_Ph), 74.1 (C-4), 74.0 (C-2), 73.2 (carborane **C**), 73.1 (6-O**C**H_2_-carborane), 71.6 (3-O**C**H_2_Ph), 71.1 (1-O**C**H_2_Ph), 71.0 (C-6) and 58.0 (carborane **C**H) ppm.

^11^B{^1^H} NMR (160.36 MHz, CDCl_3_, 25 °C): δ = −2.4, −4.3, −8.7, −11.2, and −12.7 ppm.

HRMS: *m*/*z* calcd for C_37_H_48_B_10_O_6_Na [M + Na]^+^ 721.4279; found 721.4298.

##### 2.1.2.6 6-*O*-(*o*-Carboranylmethyl)-d-mannopyranose (**1**)

This compound was synthesized from **6** (0.22 g, 0.3 mmol) and 10% Pd/C (0.22 g) according to the general procedure for deprotection of benzyl groups. This reaction gave the title compound as a white solid (0.09 g, 83%, α:β 73:27). TLC: *R*_f_: 0.70 (DCM:MeOH 5:1).

α anomer: ^1^H NMR (499.83 MHz, MeOD, 25 °C): δ = 5.03 (d, 1H, *J*_1,2_ = 1.9 Hz, H-1), 4.68 (br s, 1H, carborane C**H**), 4.06 and 4.02 (each d, each 1H, *J* = −11.0 Hz, 6-OC**H_2_**-carborane), 3.783 (dd, 1H, *J*_2,3_ = 1.6, *J*_3,4_ = 9.5 Hz, H-3), 3.781 (dd, 1H, *J*_5,6a_ = 1.9, *J*_6a,6b_ = −11.7 Hz, H-6a), 3.77 (dd, 1H, H-2), 3.73 (ddd, 1H, *J*_4,5_ = 8.6, *J*_5,6b_ = 4.2 Hz, H-5), 3.71 (dd, 1H, H-6b), 3.62 (dd, 1H, H-4) and 2.99–1.36 (br m, 10H, carborane B**H**) ppm.

^13^C{^1^H} NMR (125.69 MHz, MeOD, 25 °C): δ = 95.9 (C-1), 75.2 (carborane **C**), 73.9 (6-O**C**H_2_-carborane), 73.3 (C-2), 72.7 (C-6), 72.6 (C-2), 72.2 (C-5), 68.4 (C-4), and 60.4 (carborane **C**H) ppm.

All forms: ^11^B{^1^H} NMR (160.36 MHz, MeOD, 25 °C): δ = −2.8, −4.6, −9.0, −11.2, and −12.7 ppm.

β anomer: ^1^H NMR (499.83 MHz, MeOD, 25 °C): δ = 4.71 (d, 1H, *J*_1,2_ = 0.7 Hz, H-1), 4.68 (br s, 1H, carborane C**H**), 4.07 and 4.03 (each d, each 1H, *J* = −11.2 Hz, 6-OC**H_2_**-carborane), 3.80 (dd, 1H, *J*_2,3_ = 3.1 Hz, H-2), 3.78 (dd, 1H, *J*_5,6a_ = 5.3, *J*_6a,6b_ = −11.4 Hz, H-6a), 3.73 (dd, 1H, *J*_5,6b_ = 2.9 Hz, H-6b), 3.54 (dd, 1H, *J*_3,4_ = 9.5, *J*_4,5_ = 9.8 Hz, H-4), 3.43 (dd, 1H, H-3), 3.28 (ddd, 1H, H-5) and 2.99–1.36 (br m, 10H, carborane B**H**) ppm.

^13^C{^1^H} NMR (125.69 MHz, MeOD, 25 °C): δ = 95.6 (C-1), 77.2 (C-5), 75.3 (C-3), 75.2 (carborane **C**), 73.9 (6-O**C**H_2_-carborane), 73.0 (C-6), 72.4 (C-2), 68.1 (C-4) and 60.6 (carborane **C**H) ppm.

HRMS: *m*/*z* calcd for C_9_H_24_B_10_O_6_Na [M + Na]^+^ 361.2401; found 361.2375.

##### 2.1.2.7 1,2,3,4-Tetra-*O*-benzyl-α-d-allopyranoside (**7**)

This compound was synthesized from 1,2,3,4-tetra-*O*-benzyl-6-*O*-(*tert*-butyldimethylsilyl)-d-allopyranoside (4.68 g, 7.1 mmol) according to the general procedure for silyl group removal using HF-pyridine. This reaction gave the title compound as a white solid (2.78 g, 72%). TLC: *R*_f_: 0.32 (EtOAc:Hex 1:2).

^1^H NMR (499.83 MHz, CDCl_3_, 25 °C): δ = 7.45–7.22 (m, 20H, arom. H), 5.02 and 4.86 (each d, each 1H, *J* = −12.0 Hz, 3-OC**H_2_**Ph), 4.97 (d, 1H, *J*_1,2_ = 4.0 Hz, H-1), 4.80 and 4.57 (each d, each 1H, *J* = −12.4 Hz, 1-OC**H_2_**Ph), 4.58 and 4.42 (each d, each 1H, *J* = −11.7 Hz, 4-OC**H_2_**Ph), 4.56 and 4.54 (each d, each 1H, *J* = −13.2 Hz, 2-OC**H_2_**Ph), 4.24 (dd, 1H, *J*_2,3_ = 2.7, *J*_3,4_ = 2.7 Hz, H-3), 4.19 (ddd, 1H, *J*_4,5_ = 9.8, *J*_5,6a_ = 3.8, *J*_5,6b_ = 3.1 Hz, H-5), 3.75 (dd, 1H, *J*_6a,6b_ = −10.8 Hz, H-6a), 3.73 (dd, 1H, *J*_5,6b_ = 3.1 Hz, H-6b), 3.42 (dd, 1H, H-4) and 3.40 (dd, 1H, H-2) ppm.

^13^C{^1^H} NMR (125.69 MHz, CDCl_3_, 25 °C): δ = 139.75–127.2 (arom. C), 96.4 (C-1), 77.0 (C-2), 74.9 (C-4), 73.7 (3-O**C**H_2_Ph), 72.5 (C-3), 71.4 (2-O**C**H_2_Ph), 71.0 (4-O**C**H_2_Ph), 69.8 (1-O**C**H_2_Ph), 67.1 (C-5) and 62.2 (C-6) ppm.

HRMS: *m*/*z* calcd for C_34_H_36_O_6_Na [M + Na]^+^ 563.2410; found 563.2381.

##### 2.1.2.8 1,2,3,4-Tetra-*O*-benzyl-6-*O*-propargyl-α-d-allopyranoside (**8**)

This compound was synthesized from **7** (0.37 g, 0.7 mmol), NaH (0.03 g, 1.3 mmol), and propargyl bromide (0.09 mL, 1.0 mmol) according to the general procedure for alkylation of a free OH group. This reaction gave the title compound as a white solid (0.28 g, 77%). TLC: *R*_f_: 0.73 (EtOAc:Hex 1:2).

^1^H NMR (499.83 MHz, CDCl_3_, 25 °C): δ = 7.45–7.20 (m, 20H, arom. H), 5.00 (d, 1H, *J*_1,2_ = 3.9 Hz, H-1), 4.99 and 4.87 (each d, each 1H, *J* = −12.0 Hz, 3-OC**H_2_**Ph), 4.81 and 4.57 (each d, each 1H, *J* = −12.4 Hz, 1-OC**H_2_**Ph), 4.57 and 4.50 (each d, each 1H, *J* = −11.7 Hz, 4-OC**H_2_**Ph), 4.528 and 4.529 (each d, each 1H, *J* = −13.1 Hz, 2-OC**H_2_**Ph), 4.28 (ddd, 1H, *J*_4,5_ = 10.2, *J*_5,6a_ = 2.9, *J*_5,6b_ = 2.6 Hz, H-5), 4.19 (dd, 1H, *J*_2,3_ = 2.8, *J*_3,4_ = 2.1 Hz, H-3), 4.10 (dd, 1H, *J*_CH,CH2a_ = −2.4, *J*_CH2a,CH2b_ = −15.9 Hz, 6-OC**H_2a_**C≡CH), 4.10 (dd, 1H, *J*_CH,CH2b_ = −2.4 Hz, 6-OC**H_2b_**C≡CH), 3.87 (dd, 1H, *J*_6a,6b_ = −10.1 Hz, H-6a), 3.58 (dd, 1H, H-4), 3.53 (dd, 1H, H-6b), 3.43 (dd, 1H, H-2)and 2.35 (dd, 1H, 6-OCH_2_C≡C**H**) ppm.

^13^C{^1^H} NMR (125.69 MHz, CDCl_3_, 25 °C): δ = 139.6–127.1 (arom. C), 96.7 (C-1), 79.7 (6-OCH_2_**C**≡CH), 76.8 (C-2), 74.9 (6-OCH_2_C≡**C**H), 74.5 (C-4), 73.8 (3-O**C**H_2_Ph), 73.0 (C-3), 71.4 and 71.3 (2-O**C**H_2_Ph and 4-O**C**H_2_Ph), 69.8 (1-O**C**H_2_Ph), 68.4 (C-6), 66.4 (C-5) and 58.7 (6-O**C**H_2_C≡CH) ppm.

HRMS: *m*/*z* calcd. For C_37_H_38_O_6_Na [M + Na]^+^ 601.2566; found 601.3194.

##### 2.1.2.9 1,2,3,4-Tetra-*O*-benzyl-6-*O*-(*o*-carboranylmethyl)-α-d-allopyranoside (**9**)

This compound was synthesized from **8** (0.31 g, 0.5 mmol) and B_10_H_14_ (0.11 g, 0.9 mmol) according to the general procedure for installation of the carboranyl moiety. This reaction gave rise to the title compound as a white solid (0.25 g, 66%). TLC: *R*_f_: 0.72 (EtOAc:Hex 1:2).

^1^H NMR (499.83 MHz, CDCl_3_, 25 °C): δ = 7.44–7.21 (m, 20H, arom. H), 5.03 and 4.83 (each d, each 1H, *J* = −11.9 Hz, 3-OC**H_2_**Ph), 4.97 (d, 1H, *J*_1,2_ = 4.0 Hz, H-1), 4.78 and 4.56 (each d, each 1H, *J* = −12.4 Hz, 1-OC**H_2_**Ph), 4.58 and 4.57 (each d, each 1H, *J* = −14.1 Hz, 2-OC**H_2_**Ph), 4.56 and 4.32 (each d, each 1H, *J* = −11.8 Hz, 4-OC**H_2_**Ph), 4.27 (dd, 1H, *J*_2,3_ = 2.7, *J*_3,4_ = 2.7 Hz, H-3), 4.24 (ddd, 1H, *J*_4,5_ = 9.9, *J*_5,6a_ = 4.6, *J*_5,6b_ = 1.9 Hz, H-5), 3.89 and 3.80 (each d, each 1H, *J* = −10.6 Hz, 6-OC**H_2_**-carborane), 3.75 (br s, 1H, carborane C**H**), 3.67 (dd, 1H, *J*_6a,6b_ = −11.1 Hz, H-6a), 3.54 (dd, 1H, H-6b), 3.41 (dd, 1H, H-2), 3.32 (dd, 1H, H-4) and 2.99–0.92 (br m, 10H, carborane B**H**) ppm.

^13^C{^1^H} NMR (125.69 MHz, CDCl_3_, 25 °C): δ = 139.3–127.3 (arom. C), 96.3 (C-1), 77.4 (C-2), 74.6 (C-4), 73.8 (3-O**C**H_2_Ph), 73.0 (carborane **C**), 72.9 (6-O**C**H_2_-carborane), 72.2 (C-3), 71.5 (2-O**C**H_2_Ph), 71.4 (C-6), 70.5 (4-O**C**H_2_Ph), 69.8 (1-O**C**H_2_Ph), 66.8 (C-5) and 57.6 (carborane **C**H) ppm.

^11^B{^1^H} NMR (160.36 MHz, CDCl_3_, 25 °C): δ = −3.2, −5.0, −9.3, −11.9, and −13.4 ppm.

HRMS: *m*/*z* calcd for C_37_H_48_B_10_O_6_Na [M + Na]^+^ 721.4279; found 721.4239.

##### 2.1.2.10 6-*O*-(*o*-Carboranylmethyl)-d-allopyranose (**2**) and 6-*O*-(*o*-Carboranylmethyl)-d-allofuranose

This compound was synthesized from **9** (0.28 g, 0.4 mmol) and 10% Pd/C (0.28 g) according to the general procedure for deprotection of benzyl groups. This reaction gave the title compound as a white solid (0.11 g, 80%). TLC: *R*_f_: 0.69 (DCM:MeOH 5:1). Product distribution: α-pyranose:β-pyranose:α-furanose:β-furanose 28:63:3:6.

Pyranose Forms

α anomer: ^1^H NMR (499.83 MHz, MeOD, 25 °C): δ = 5.01 (d, 1H, *J*_1,2_ = 3.7 Hz, H-1), 4.60 (br s, 1H, carborane C**H**), 4.06 (dd, 1H, *J*_2,3_ = 2.6, *J*_3,4_ = 2.8 Hz, H-3), 4.03 and 4.02 (each d, each 1H, *J* = −9.9 Hz, 6-OC**H_2_**-carborane), 3.95 (ddd, 1H, *J*_4,5_ = 10.2, *J*_5,6a_ = 1.7, *J*_5,6b_ = 4.9 Hz, H-5), 3.77 (dd, 1H, *J*_6a,6b_ = −11.1 Hz, H-6a), 3.76 (dd, 1H, H-6b), 3.50 (dd, 1H, H-2), 3.46 (dd, 1H, H-4) and 2.99–1.36 (br m, 10H, carborane B**H**) ppm.

^13^C{^1^H} NMR (125.69 MHz, MeOD, 25 °C): δ = 95.2 (C-1), 75.3 (carborane **C**), 74.1 (C-3), 73.8 (6-O**C**H_2_-carborane), 72.7 (C-6), 69.0 (C-2), 68.1 (C-4), 68.0 (C-5) and 60.6 (carborane **C**H) ppm.

β anomer: ^1^H NMR (499.83 MHz, MeOD, 25 °C): δ = 4.79 (d, 1H, *J*_1,2_ = 8.0 Hz, H-1), 4.63 (br s, 1H, carborane C**H**), 4.04 and 4.01 (each d, each 1H, *J* = −10.8 Hz, 6-OC**H_2_**-carborane), 4.04 (dd, 1H, *J*_2,3_ = 3.3, *J*_3,4_ = 2.8 Hz, H-3), 3.77 (ddd, 1H, *J*_4,5_ = 9.8, *J*_5,6a_ = 1.9, *J*_5,6b_ = 5.4 Hz, H-5), 3.76 (dd, 1H, *J*_6a,6b_ = −11.3 Hz, H-6a), 3.68 (dd, 1H, H-6b), 3.47 (dd, 1H, H-4), 3.22 (dd, 1H, H-2) and 2.99–1.36 (br m, 10H, carborane B**H**) ppm.

^13^C{^1^H} NMR (125.69 MHz, MeOD, 25 °C): δ = 95.4 (C-1), 75.3 (carborane **C**), 75.4 (C-5), 73.9 (6-O**C**H_2_-carborane), 73.4 (C-2), 72.8 (C-6 and C-3), 68.7 (C-4), and 60.6 (carborane **C**H) ppm.

Furanose Forms

α anomer: ^1^H NMR (499.83 MHz, MeOD, 25 °C): δ = 5.20 (d, 1H, *J*_1,2_ = 4.1 Hz, H-1), 4.68 (br s, 1H, carborane C**H**), 4.08 (dd, 1H, *J*_2,3_ = 4.0, *J*_3,4_ = 5.3 Hz, H-3), 4.04 and 4.02 (each d, each 1H, *J* = −10.6 Hz, 6-OC**H_2_**-carborane), 3.96 (dd, 1H, *J*_4,5_ = 8.7 Hz, H-4), 3.94 (dd, 1H, H-2), 3.71 (ddd, 1H, *J*_5,6a_ = 4.1, *J*_5,6b_ = 6.2 Hz, H-5), 3.62 (dd, 1H, *J*_6a,6b_ = −10.3 Hz, H-6a), 3.55 (dd, 1H, H-6b) and 2.99–1.36 (br m, 10H, carborane B**H**) ppm.

β anomer: ^1^H NMR (499.83 MHz, MeOD, 25 °C): δ = 5.11 (d, 1H, *J*_1,2_ = 1.7 Hz, H-1), 4.68 (br s, 1H, carborane C**H**), 4.29 (dd, 1H, *J*_2,3_ = 4.8, *J*_3,4_ = 5.8 Hz, H-3), 4.04 and 4.01 (each d, each 1H, *J* = −11.0 Hz, 6-OC**H_2_**-carborane), 3.821 (dd, 1H, H-2), 3.820 (dd, 1H, *J*_4,5_ = 5.7 Hz, H-4), 3.78 (ddd, 1H, *J*_5,6a_ = 4.6, *J*_5,6b_ = 6.5 Hz, H-5), 3.67 (dd, 1H, *J*_6a,6b_ = −10.6 Hz, H-6a), 3.58 (dd, 1H, H-6b) and 2.99–1.36 (br m, 10H, carborane B**H**) ppm.

All forms: ^11^B{^1^H} NMR (160.36 MHz, MeOD, 25 °C): δ = −2.7, −4.6, −8.9, −11.1, −12.2, and −12.6 ppm.

HRMS: *m*/*z* calcd for C_9_H_24_B_10_O_6_Na [M + Na]^+^ 361.2401; found 361.2440.

##### 2.1.2.11 1,2:3,4-Bis-*O*-(isopropylidene)-6-*O*-propargyl-α-d-galactopyranoside (**10**)

This compound was synthesized from 1,2:3,4-bis-*O*-(isopropylidene)-α-d-galactopyranoside (0.54 g, 2.1 mmol), NaH (0.19 g, 4.76 mmol), and propargyl bromide (0.55 g, 3.73 mmol) according to the general procedure for alkylation of a free OH group. This reaction gave the title compound as a white solid (0.43 g, 70%). TLC: *R*_f_: 0.34 (EtOAc:Hex 1:4).

^1^H NMR (499.83 MHz, CDCl_3_, 25 °C): δ = 5.54 (d, 1H, *J*_1,2_ = 5.1 Hz, H-1), 4.61 (dd, 1H, *J*_2,3_ = 2.4, *J*_3,4_ = 7.9 Hz, H-3), 4.32 (dd, 1H, H-2), 4.26 (dd, 1H, *J*_4,5_ = 2.0 Hz, H-4), 4.25 (dd, 1H, *J*_CH,CH2a_ = −2.4, *J*_CH2a,CH2b_ = −15.9 Hz, 6-OC**H_2a_**C≡CH), 4.20 (dd, 1H, *J*_CH,CH2b_ = −2.4 Hz, 6-OC**H_2b_**C≡CH), 4.00 (ddd, 1H, *J*_5,6a_ = 5.3, *J*_5,6b_ = 7.1 Hz, H-5), 3.77 (dd, 1H, *J*_6a,6b_ = −10.1 Hz, H-6a), 3.67 (dd, 1H, H-6b), 2.43 (dd, 1H, 6-OCH_2b_C≡C**H**), 1.54, 1.45, 1.34 and 1.33 (each s, each 3H, 1,2-C(CH_3_)_2_ and 3,4-C(CH_3_)_2_) ppm.

^13^C{^1^H} NMR (125.69 MHz, CDCl_3_, 25 °C): δ = 109.5 (1,2-^q^C), 108.8 (3,4-^q^C), 96.5 (C-1), 79.8 (6-OCH_2_**C**≡CH), 74.7 (6-OCH_2_C≡**C**H), 71.3 (C-4), 70.8 (C-3), 70.6 (C-2), 68.9 (C-6), 66.9 (C-5), 58.7 (6-O**C**H_2_C≡CH), 26.2, 26.1, 25.1 and 24.6 (1,2-C(**C**H_3_)_2_ and 3,4-C(**C**H_3_)_2_) ppm.

HRMS: *m*/*z* calcd for C_15_H_22_O_6_Na [M + Na]^+^ 298.1416; found 298.1458.

##### 2.1.2.12 1,2:3,4-Bis-*O*-(isopropylidene)-6-*O*-carboranylmethyl-α-d-galactopyranoside (**11**)

This compound was synthesized from **10** (0.43 g, 1.4 mmol) and B_10_H_14_ (0.30 g, 2.4 mmol) according to the general procedure for installation of the carboranyl moiety. This reaction gave the title compound as a white solid (0.37 g, 62%). TLC: *R*_f_: 0.43 (EtOAc:Hex 1:4).

^1^H NMR (499.83 MHz, CDCl_3_, 25 °C): δ = 5.50 (d, 1H, *J*_1,2_ = 5.0 Hz, H-1), 4.60 (dd, 1H, *J*_2,3_ = 2.5, *J*_3,4_ = 7.9 Hz, H-3), 4.31 (dd, 1H, H-2), 4.16 (dd, 1H, *J*_4,5_ = 1.9 Hz, H-4), 4.10 (br s, 1H, carborane-C**H**), 4.04 and 3.93 (each d, each 1H, *J* = −10.8 Hz, 6-OC**H_2_**-carborane), 3.91 (ddd, 1H, *J*_5,6a_ = 4.7, *J*_5,6b_ = 7.3 Hz, H-5), 3.70 (dd, 1H, *J*_6a,6b_ = −10.8 Hz, H-6a), 3.61 (dd, 1H, H-6b), 3.16–1.10 (br m, 10H, carborane-B**H**), 1.53, 1.43, 1.33 and 1.32 (each s, each 3H, 1,2-C(CH_3_)_2_ and 3,4-C(CH_3_)_2_) ppm.

^13^C{^1^H} NMR (125.69 MHz, CDCl_3_, 25 °C): δ = 109.7 (1,2-^q^C), 108.9 (3,4-^q^C), 96.4 (C-1), 74.7 (carborane-**C**), 72.7 (6-O**C**H_2_-carborane), 71.2 (C-6), 71.1 (C-4), 70.8 (C-3), 70.6 (C-2), 67.4 (C-5), 57.8 (carborane-**C**H), 26.2, 26.1, 25.0, and 24.5 (1,2-C(**C**H_3_)_2_ and 3,4-C(**C**H_3_)_2_) ppm.

^11^B{^1^H} NMR (160.36 MHz, CDCl_3_, 25 °C): δ = −3.1, −5.0, −9.3, −11.8, and −13.4 ppm.

HRMS: *m*/*z* calcd for C_15_H_32_B_10_O_6_Na [M + Na]^+^ 441.3027; found 441.3055.

##### 2.1.2.13 6-*O*-(*o*-Carboranylmethyl)-d-galactopyranose (3) and 6-*O*-Carboranylmethyl-d-galactofuranose

This compound was synthesized from **11** (0.21 g, 0.50 mmol) according to the general procedure for removal of acetal protecting groups. This reaction gave the title compound as a white solid (0.16 g, 97%). TLC: *R*_f_: 0.64 (EtOAc:MeOH 5:1). Product distribution: α-pyranose:β-pyranose:α-furanose:β-furanose 40:47:8:5.

Pyranose Forms

α anomer: ^1^H NMR (499.83 MHz, MeOD, 25 °C): δ = 5.14 (d, 1H, *J*_1,2_ = 3.9 Hz, H-1), 4.60 (br s, 1H, carborane C**H**), 4.15 (ddd, 1H, *J*_4,5_ = 1.3, *J*_5,6a_ = 5.5, *J*_5,6b_ = 6.9 Hz, H-5), 4.04 and 4.00 (each d, each 1H, *J* = −11.1 Hz, 6-OC**H_2_**-carborane), 3.84 (dd, 1H, *J*_3,4_ = 3.3 Hz, H-4), 3.78 (dd, 1H, *J*_2,3_ = 10.1 Hz, H-3), 3.73 (dd, 1H, H-2), 3.727 (dd, 1H, *J*_6a,6b_ = −10.3 Hz, H-6a), 3.63 (dd, 1H, H-6b), and 3.01–1.43 (br m, 10H, carborane B**H**) ppm.

^13^C{^1^H} NMR (125.69 MHz, MeOD, 25 °C): δ = 94.2 (C-1), 75.1 (carborane **C**), 73.7 (6-O**C**H_2_-carborane), 72.6 (C-6), 71.1 (C-3), 71.0 (C-4), 70.3 (C-2), 70.0 (C-5) and 60.7 (carborane **C**H) ppm.

β anomer: ^1^H NMR (499.83 MHz, MeOD, 25 °C): δ = 4.44 (d, 1H, *J*_1,2_ = 7.6 Hz, H-1), 4.63 (br s, 1H, carborane C**H**), 4.05 and 4.01 (each d, each 1H, *J* = −11.2 Hz, 6-OC**H_2_**-carborane), 3.78 (dd, 1H, *J*_3,4_ = 3.3, *J*_4,5_ = 1.2 Hz, H-4), 3.74 (dd, 1H, *J*_5,6a_ = 5.2, *J*_6a,6b_ = −10.2 Hz, H-6a), 3.69 (ddd, 1H, *J*_5,6b_ = 6.9 Hz, H-5), 3.68 (dd, 1H, H-6a), 3.48 (dd, 1H, H-3), 3.46 (dd, 1H, H-2) and 3.01–1.43 (br m, 10H, carborane B**H**) ppm.

^13^C{^1^H} NMR (125.69 MHz, MeOD, 25 °C): δ = 98.7 (C-1), 75.1 (carborane **C**), 74.8 (C-3 and C-5), 73.7 (C-2), 73.66 (6-O**C**H_2_-carborane), 72.3 (C-6), 70.4 (C-4) and 60.8 (carborane **C**H) ppm.

Furanose Forms

α anomer: ^1^H NMR (499.83 MHz, MeOD, 25 °C): δ = 5.12 (d, 1H, *J*_1,2_ = 2.7 Hz, H-1), 4.66 (br s, 1H, carborane C**H**), 4.03 (dd, 1H, *J*_2,3_ = 4.3, *J*_3,4_ = 6.2 Hz, H-3), 4.03 and 4.00 (each d, each 1H, *J* = −11.0 Hz, 6-OC**H_2_**-carborane), 4.00 (dd, 1H, *J*_4,5_ = 3.1 Hz, H-4), 3.90 (dd, 1H, H-2), 3.82 (ddd, 1H, *J*_5,6a_ = 5.3, *J*_5,6b_ = 6.9 Hz, H-5), 3.63 (dd, 1H, *J*_6a,6b_ = −10.1 Hz, H-6a), 3.58 (dd, 1H, H-6b) and 3.01–1.43 (br m, 10H, carborane B**H**) ppm.

β anomer: ^1^H NMR (499.83 MHz, MeOD, 25 °C): δ = 5.17 (d, 1H, *J*_1,2_ = 4.4 Hz, H-1), 4.66 (br s, 1H, carborane C**H**), 4.14 (ddd, 1H, *J*_4,5_ = 1.1, *J*_5,6a_ = 5.2, *J*_5,6b_ = 6.2 Hz, H-5), 4.03 and 4.01 (each d, each 1H, *J* = −11.2 Hz, 6-OC**H_2_**-carborane), 3.91 (dd, 1H, *J*_2,3_ = 6.6 Hz, H-2), 3.77 (dd, 1H, *J*_3,4_ = 4.0 Hz, H-4), 3.76 (dd, 1H, H-3), 3.62 (dd, 1H, *J*_6a,6b_ = −9.9 Hz, H-6a), 3.54 (dd, 1H, H-6b) and 3.01–1.43 (br m, 10H, carborane B**H**) ppm.

All forms: ^11^B{^1^H} NMR (160.36 MHz, MeOD, 25 °C): δ = −2.6, −4.5, −8.9, −11.1, −12.2, and −12.7 ppm.

HRMS: *m*/*z* calcd for C_9_H_24_B_10_O_6_Na [M + Na]^+^ 361.2401; found 361.2462.

### 2.2 In Vitro Assessment Protocols

CAL 27 (ATCC CRL-2095) cells used in in vitro experiments were acquired from ATCC (Manassas, VA, USA) and cultured in Dulbecco’s modified Eagle’s medium (DMEM) supplemented with l-glutamine (2.0 mM), heat-inactivated fetal bovine serum (10%), and penicillin (50 U/mL)–streptomycin (50 μg/mL) at 37 °C with 5% CO_2_ and 95% relative humidity. The GLUT1 affinity studies, cellular uptake, as well as the cytotoxicity studies followed the protocols reported in our earlier work and will only be briefly described here.^16,20^

#### 2.2.1 GLUT1 Affinity Studies

The CAL 27 cells were incubated at r.t. for 5 min with the glycoconjugates **1–3** (0.1–1800 μM) containing [14C]-d-glucose (1.8 μM, 0.1 mCi/mL) in glucose-free HBSS (Hanks’ balanced salt solution). The reaction was quenched with ice-cold buffer, followed by two times washing of the cells, lysis with 250 μL of 0.1 M NaOH, and mixing with 1.0 mL of emulsifier safe cocktail (PerkinElmer, Waltham, MA, USA). The IC_50_ values were obtained by nonlinear regression analysis.

#### 2.2.2 Cellular Uptake Studies

CAL 27 cells were pre-incubated after which the glycoconjugates **1–3** were added in a concentration range of 10–400 μM in 250 μL of glucose-free HBSS. They were further incubated for 5, 30, and 120 min at r.t. The reaction was quenched with adding ice-cold buffer followed by washing of the cells. The cell lysate from four wells was combined and centrifuged at 4 °C. Supernatant (800 μL) from each sample was collected and digested in 1.0 mL of HNO_3_ for 24 h. Milli-Q water was added to obtain 10 mL of the sample, and the boron amount was measured using ICP-MS. The measurements were performed in triplicate. The data analysis was performed with PerkinElmer Syngistix Data Analysis Software, and the statistical analyses was done using GraphPad Prism v.5.03.

#### 2.2.3 Cytotoxicity Experiments

CAL 27 cells were seeded at a density of 5000 cells per well in a white-walled 96-well plate and incubated overnight. The cells were incubated in cell culture media containing glycoconjugates **1–3** at concentrations of 5, 25, 50, 125, and 250 μM for 24 h at 37 °C, 5% CO_2_ atmosphere, and 95% relative humidity. Each concentration was done in triplicate. After 24 h, media was removed, and the cells were washed twice with 1 × DPBS (Dulbecco’s Phosphate Buffered Saline) (pH 7.4). To assess cell viability, an equal volume of 1 × HBSS and CellTiter-Glo reagent (50 μL each) was added to all the wells, and they were left at r.t. in the dark for 10 min. The luminescent signal from viable cells was measured using the Synergy H1 Hybrid multimode microplate reader (BioTek, Winooski, VT, USA). After luminescence reading, the total protein content in each sample was quantified using the Pierce colorimetric bicinchoninic acid (BCA) protein assay (Thermo Fisher Scientific, Waltham, MA, USA). Briefly, a sample of 25 μL from each well was transferred to a separate transparent 96-well plate. BCA working reagent (200 μL) was then added to each well, and the plates were wrapped with aluminum foil and incubated for another 30 min at 37 °C. The absorbance was recorded at 562 nm using the microplate reader. The protein content was calculated using the BSA standard curve (concentration range: 0–2000 μg/mL) and used to normalize cell viability data.

### 2.3 Molecular Modeling

The initial geometries of the ligands were optimized to a local minimum at the DFT level using the dispersion-corrected hybrid Tao–Perdew–Scuseria–Staroverov functional TPSSh-D3(BJ),^29−31^ with the doubly polarized triple-ζ basis set def2-TZVPP.^32^ Partial atomic charges were computed using the restrained electrostatic potential (RESP) protocol.^33^ For the RESP charge calculation, the molecule was divided into two parts, with one part consisting of the carborane and a linking carbon atom and the other part comprising the sugar. Partial charges of hydrogen atoms bonded to the same carbon atom were constrained to be equal. The geometry optimizations were performed with Turbomole 7.6^34,35^ and the RESP calculations with NWChem 6.8.1.^36^

Molecular docking studies were performed using AutoDock 4.2.6.^37,38^ All rotatable bonds in the carborane part were set to nonrotatable (inactive). For docking, the number of torsional degrees of freedom was set to 8 (torsdof 8) for the pyranoses and to 9 (torsdof 9) for the furanoses. The docking studies were performed using the XylE inward-open 4QIQ(39) and outward-open 6N3I(40) PDB structures. The XylE protein structures were mutated using PyMOL, changing GLN-415 to ASN-415. The most probable rotamer, that is, the one with the least clashes with surrounding amino acids, as suggested by PyMOL, was used. Each protein was prepared by removing the ligand and other superfluous small molecules (Zn for 4QIQ), adding hydrogens, merging them, and then computing Gasteiger partial charges. For all proteins, a grid of size 46 × 56 × 60 was used, with a grid spacing value of 0.375. The grid center was in the middle of the protein cavity for the grid box to cover the binding site. During docking, the protein was kept rigid and only ligand torsional angles changed. For each ligand, 2000 independent search runs, each with max 2.5 million energy evaluations and a population size of 150 with max 27,000 generations, were performed using the default settings of the Lamarckian genetic algorithm (LGA), that is, a mutation rate of 0.02 and a crossover rate of 0.8, with one top individual surviving to the next generation. Conformations were clustered (ranked) with a cluster RMS of 2.0 Å.

Parameters for boron, missing from the standard distribution of Autodock, were added to the parameter file: R 2.285, Rii 4.57, epsilon 0.179, vol 49.9744; other parameters were set to their corresponding carbon values. R and epsilon were taken from Oda et al.,^41^ as reproduced by Couto et al.,^42^ and were used to calculate Rii and vol. The complete parameter definition was thus: atom_par B 4.57 0.179 49.9744–0.00143 0.0 0.0 0 −1 −1 0 # boron for carborane.

## 3 Results

### 3.1 Synthesis of Glycoconjugate-Based Delivery Agents

**Scheme 1:**
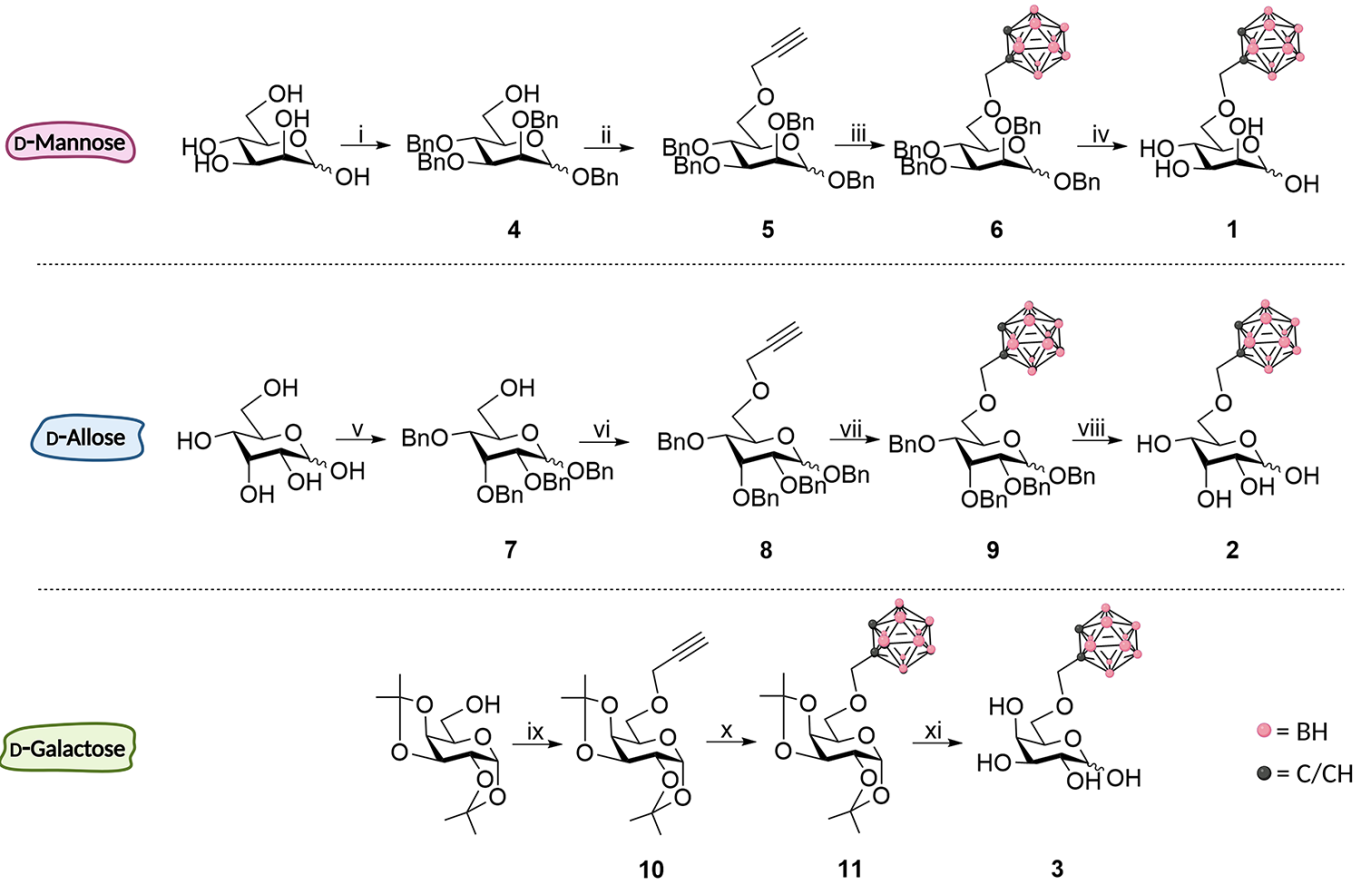
Reaction Routes and Conditions Employed in the Synthesis of Glycoconjugates **1** (Top), **2** (Middle), and **3** (Bottom) Top: (i) (1) *tert*-butyldimethylsilyl chloride (TBDMSCl), pyridine, r.t., 2 h, 68%; (2) BnBr, NaH, DMF, 0 °C → r.t., 2 h, 80%; (3) HF·pyridine, THF, 0 °C → r.t., 18 h, 60%; (ii) propargyl bromide, NaH, DMF, r.t., 2 h, 70%; (iii) (1) B_10_H_14_, MeCN, 60 °C, 1 h; (2) **5**, toluene, 80 °C, 16 h, 53%; (iv) H_2_, 10% Pd/C, EtOAc:MeOH 7:1, 4 bar, r.t., 6 h, 83%. Middle: (v) (1) TBDMSCl, pyridine, r.t., 4 h, 78%; (2) BnBr, NaH, DMF, 0 °C → r.t., 3 h, 88%; (3) HF·pyridine, THF, 0 °C → r.t., 18 h, 72%; (vi) propargyl bromide, NaH, DMF, r.t., 2 h, 77%; (vii) (1) B_10_H_14_, MeCN, 60 °C, 1 h; (2) **8**, toluene, 80 °C, 16 h, 66%; (viii) H_2_, 10% Pd/C, EtOAc:MeOH 7:1, 4 bar, r.t., 4 h, 80%. Bottom: (ix) NaH, propargyl bromide, DMF, r.t., 16 h, 70%; (x) (1) B_10_H_14_, MeCN, 60 °C, 1 h; (2) **10**, toluene, 80 °C, 16 h, 62%; (xi) trifluoroacetic acid (TFA)/H_2_O/Et_2_O (2:1:2), r.t., 24 h, 97%.

As mentioned above, our goal was to incorporate an *ortho*-carboranylmethyl substituent in position 6 of the targeted epimers. The stereochemical configuration in the carbohydrate cores was implemented as a base for the design of synthetic sequences leading to glycoconjugates **1–3**. While the synthesis does follow standard protecting group strategies often employed in carbohydrate chemistry, a few factors need to be taken into account when attempting to install a carborane (*closo*-dicarbadodecaborane(12)) moiety.^43^ First, our selected conjugation reaction between the decaborane complex [B_10_H_12_(MeCN)_2_] and a terminal alkyne does not tolerate the presence of free hydroxyl groups.^44^ Second, the *ortho*-carborane cluster is known to be base-labile and harsh basic conditions need to be avoided during deprotection sequences.^44^ With these factors in mind, two synthetic pathways were devised. Glycoconjugates **1** and **2** were synthesized in a similar fashion to that previously reported for the d-Glc analogue,^16^ while for glycoconjugate **3**, a shorter synthetic sequence employing isopropylidene protecting groups was devised in accordance with a previous report in the field.^45,46^ A summary of the synthetic routes is supplied in Scheme 1.

In short, the synthesis of glycoconjugates **1** and **2** was initiated by the selective protection of the 6-OH group. This can be achieved with a bulky substituent, here a *tert*-butyldimethylsilyl ether was installed in yields ranging from 68 to 78%.^47^ The remaining hydroxyl groups were benzylated in good yields,^48^ followed by cleaving of the temporary protected 6-OTBDMS group with a HF·pyridine complex.^49^ The newly demasked 6-OH group was deprotonated with NaH, and the corresponding alkoxide ion was reacted with propargyl bromide to introduce the conjugation site for the [B_10_H_12_(MeCN)_2_] complex. The [B_10_H_12_(MeCN)_2_] complex was generated by gentle heating of decaborane in dry acetonitrile, and the carboranyl installation was successfully completed by the addition of the protected carbohydrate derivative (**5** or **8**) bearing the terminal alkyne handle in dry toluene. The 53–66% yields observed in the carboranyl installation are typical for reactions of this type.^44^ The final hydrogenolysis of benzyl groups was performed in an autoclave using 10% Pd/C as the catalyst.^50^ This led to the formation of **1** and **2** in yields ranging from 80 to 83%. The overall efficiencies of these synthetic routes were thus 10–20% over six steps.

Glycoconjugate **3** can be accessed through a shorter synthetic route starting from commercially available 1,2:3,4-bis-*O*-isopropylidene-α-d-galactopyranose. Similar strategies as those earlier reported for the synthesis of glycoconjugate **3** were employed.^45,46^ Propargylation and carboranyl installation with B_10_H_12_(MeCN)_2_ proceeded in similar yields to those observed in the synthetic routes leading to **1** and **2**. The deprotection, performed under acidic conditions, gave glycoconjugate **3** in an excellent yield, improving upon the deprotection yield reported in the literature for **3**.^46^ The overall efficiency of this synthetic route was 42% over three steps. All three synthetic routes were relatively short and displayed overall yields in the 10–42% range. They employed commercially available and affordable reagents and can therefore be considered to be cost-effective. Further optimization of work processes would however be required in order to supply materials for clinical studies.

### 3.2 Structural Characterization of Synthesized Compounds

**Figure 3:**
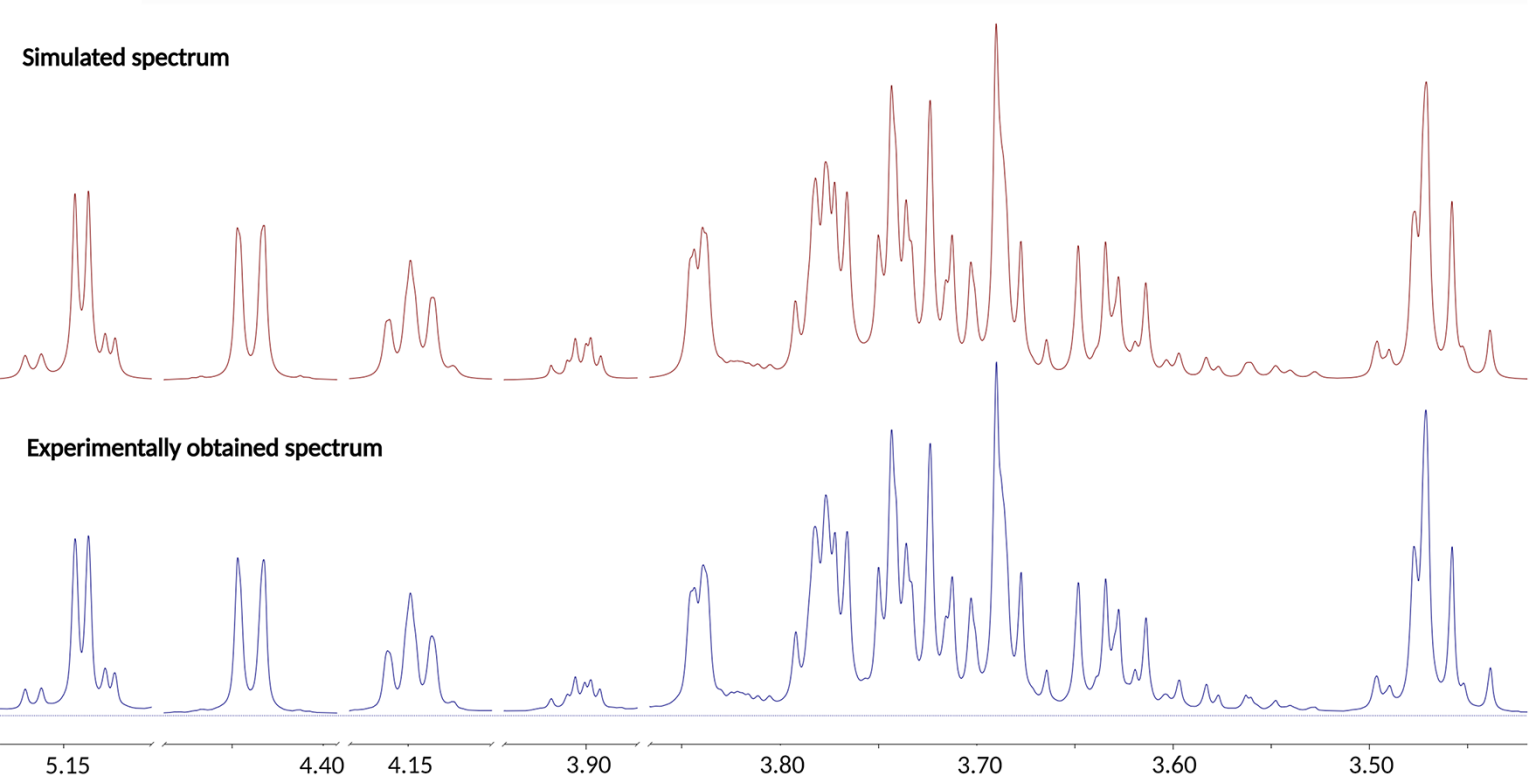
NMR spectral simulation with the ChemAdder software is showcased for selected parts of the 5.20–3.40 ppm range using glycoconjugate **3** as an example. Top: Simulated spectrum. Bottom: Experimentally obtained spectrum.

All key intermediates and final compounds were initially characterized by a conventional set of 1D- and 2D-NMR experiments and high-resolution mass spectrometry (HRMS). The purity of glycoconjugates **1–3** was confirmed by qNMR-techniques employing maleic acid as an internal standard. Apart from the purity assessment, special emphasis was placed on characterization of NMR spectra. This was considered important because mutarotation is a naturally occurring phenomenon in carbohydrates leading to the possible formation of furanose and pyranose forms as both α- and β-anomers. These different forms and anomeric ratios may impact how the substrates interact with the transporters in a biological environment, and thus, having information on the distribution between these can be useful. To this end, we initially employed the following 1D-experiments: ^1^H, 1D-TOCSY, ^13^C{^1^H} and ^11^B{H}, and 2D-experiments: COSY, ed-HSQC, and HMBC. Especially the use of 1D-TOCSY proved critical in the current study.^47,51^ With the aid of 1D-TOCSY, we were able to analyze the different spin systems overlapping in the ^1^H NMR spectra. This provided us with a good base for a detailed analysis of the spectra with the ChemAdder program.^52,53^ Through the use of ChemAdder, the spectra were simulated and fitted with the experimentally derived ones. The solid match between the simulated and experimental ^1^H NMR spectra resulted in access to information such as accurate chemical shifts, coupling constants, and distribution between the forms/anomers present in solution. An example of the NMR spectroscopic characterization studies of glycoconjugate **3** is provided in Figure 3.

For glycoconjugate **1**, only the pyranose forms were visible in the spectra, whereas for glycoconjugates **2** and **3**, small amounts of signals resembling furanoses could likewise be found. The ratio between α- and β-anomers was also largely affected by the stereochemistry of the carbohydrate core. In the d-Glc analogue, **6-oCb-Glc**, and for glycoconjugate **1**, the α-pyranose form dominates (α:β ratio of approx. 1.5:1 for **6-oCb-Glc** and 3:1 for the d-Man glycoconjugate, respectively). In d-Gal, an equal distribution between the anomers is observed, and in d-All, the β-pyranose form dominates (α:β approx. 1:2).

### 3.3 Assessing the Transporter Targeting Capabilities of Delivery Agents

For GLUT1-targeting agents to be viable in BNCT, they need to be able to compete for the hexose transporters even with the high blood concentrations of d-Glc. In our earlier work, we have proven the competitive advantage of glucoconjugates through an experimental *cis*-inhibition assay and analyzed the interactions taking place between the ligands and the GLUT1 transporter through molecular docking studies.^16,20,21^ Here, similar assessment protocols were employed.

The interactions between **1–3** and the carbohydrate recognition domain of GLUT1 were assessed through docking studies employing our existing GLUT1 model.^16,20,21^ Our GLUT1 model is derived from the inside open (PDB ID 4QIQ)^39^ and outside open (PDB ID 6N3I)^40^ crystal structures reported for a d-xylose-proton symporter (29% sequence identity, 49% similarity to GLUT1)^54^ through virtual mutation of GLN415 in the symporter in order to match the ASN found in the carbohydrate recognition domain of GLUT1.^55,56^ Glycoconjugates **1–3** were found to interact more strongly with the transporter than d-Glc which indicates that their targeting capabilities may be superior compared to the natural substrate (see mean binding energies in Supporting Information, Table S1). The mean binding energies for the three glycoconjugates were all within the 1 kcal/mol error limit (0.23 kcal/mol for the outside open conformation, 0.16 kcal/mol for the inside open conformation) of the computational methods employed, and therefore, details on which of these would be the best could not be deduced based on the computational modeling data. The computational model did however suggest that the pyranose forms bind more strongly to the carbohydrate recognition domain of GLUT1 than the furanose forms (see Supporting Information, Table S2). Therefore, monosaccharides in which the furanose form dominates might be inferior to ones in which the pyranose form dominates, although without experimental evidence to support the modeling data definite conclusions cannot be drawn. Nevertheless, in glycoconjugates **2** and **3**, the furanose forms are present in minor amounts (9–13%) and would not be expected to cause serious concerns in this regard.

**Figure 4:**
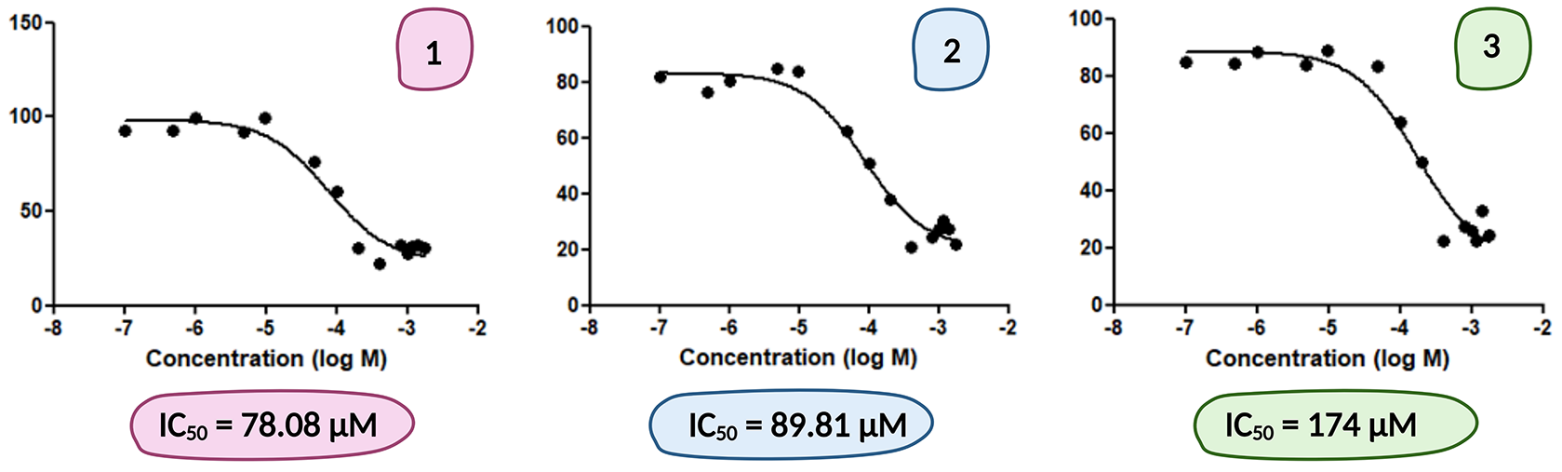
Inhibition of [^14^C]-d-glucose uptake by glycoconjugates **1–3** in the CAL 27 cell line is showcased and the IC_50_ values provided.

In the *cis*-inhibition assay employed, glycoconjugates **1–3** were forced to compete for the transporter against radiolabeled d-Glc. This assay provides essential information on the targeting capabilities of the delivery agents on a molecular biology level. In more detail, the inhibition of d-Glc uptake at low concentrations was taken as a sign of promising transporter targeting capabilities. In this regard, the IC_50_ values reported represent the concentrations at which the glycoconjugates inhibit 50% of the radiolabeled d-Glc uptake. These studies were performed on the CAL 27 cell line, which is an oral adenosquamous carcinoma cell line^23,28^ of human origin and thus relevant from the BNCT treatment perspective. In addition, we have previously determined the GLUT1-expression levels, verified the GLUT1 function, and used it as a model for comparison of GLUT1-targeting agents.^16^ Our current hit molecule, **6-oCb-Glc**, has an IC_50_ value of 43.96 μM, while the nonradiolabeled control d-Glc has an IC_50_ value exceeding 1 mM in the CAL 27 cell line.^16^ The IC_50_ values of the glycoconjugates **1–3** are summarized in Figure 4. None of the glycoconjugates **1–3** are able to match the GLUT1 affinity observed for **6-oCb-Glc**. It is important to not over-interpret these results as they reflect upon the competition for transporters targeted by d-Glc. If glycoconjugates **1–3** display different hexose transporter targeting profiles than d-Glc, information obtained through the employed assay does not necessarily account for such factors. Nevertheless, the results still indicate that the glucose transporter targeting capabilities are superior to that of the natural substrate.

### 3.4 Determining the Potential of Delivery Agents In Vitro

In order to determine whether delivery agents display sufficient potential to warrant future in vivo studies, we have studied their cytotoxicity and boron delivery capacity. Both are important factors for eventual translation into more detailed preclinical and clinical assessment studies. One of the important prospects of BNCT is that nontoxic delivery agents can be employed. Thereby, harmful side effects can be limited compared to conventional chemotherapy which employs toxic cancer therapeutics. In order to reach the required cellular boron concentrations, considerable amounts of delivery agents are used in clinical treatments. For example, the most commonly employed delivery agent BPA is administered intravenously at a dose of roughly 250–400 mg/kg of patient weight.^57^ While the amounts are bound to be different depending on the efficiency of delivery agents and strategies employed, it nevertheless underlines why a low systemic toxicity is highly beneficial for delivery agents in BNCT.

**Figure 5:**
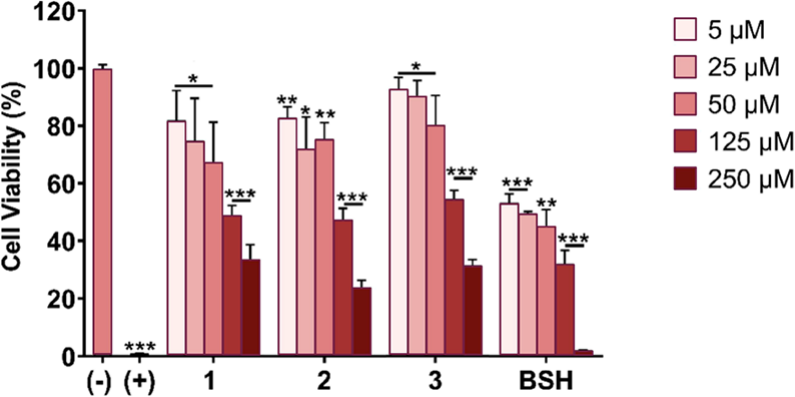
Cytotoxicity studies in the CAL 27 cell line after incubation with cell culture medium (negative control), 1% v/v Triton X-100 (positive control), glycoconjugates **1–3**, and BSH at concentrations of 5, 25, 50, 125, and 250 μM (*n* = 3–4) at the 24 h time point. The comparative cytotoxicity data for BSH is from one of our earlier studies in the same cell line.^21^ The statistical significance was analyzed using an unpaired Student’s *t*-test, where the significance was set at *p < 0.05, **p < 0.01, and ***p < 0.001.

Here, we performed a cytotoxicity evaluation in the squamous cell carcinoma CAL 27 cell line. The toxicity profiles of glycoconjugates **1–3** were assessed over the 5–250 μM concentration range in order to be able to compare the results to our earlier work. The preclinical and clinical administration doses would be expected to fall within this concentration range. While we have earlier studied the toxicity of delivery agents at multiple time points, we have concluded that the 24 h time point is sufficient for preliminary evaluation. The results from the cytotoxicity assay are summarized in Figure 5. In addition to glycoconjugates **1–3**, a complete growth medium was used as a negative control (for cytotoxicity), 1% v/v Triton X-100 as a positive control (for cytotoxicity), and BSH as the reference of a clinically employed boron delivery agent.^21^ Overall, glycoconjugates **1–3** were found to be less toxic than BSH across the concentration range, indicating that their in vitro toxicity profiles are more amenable, and their use in BNCT is not precluded.

**Figure 6:**
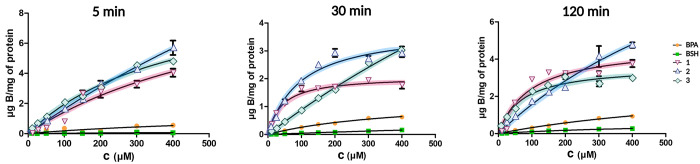
Boron delivery capacity assessed by ICP-MS determination of elemental boron in digested CAL 27 cells after incubation with glycoconjugates **1–3**, BPA, or BSH in the concentration range of 10–400 μM over 5, 30, and 120 min.

As a last measure, we sought to determine the boron delivery capacity of glycoconjugates **1–3**. This is one of the key aspects of the development of improved delivery agents for BNCT. Therefore, we considered it important to include clinically employed delivery agents (BPA and BSH) in the study to have a solid reference to compare the results to. It should be noted that these two agents enter the cells via different mechanisms than the hexose transporters targeted in our approach. BPA is a LAT1-targeting delivery agent, and BSH enters the cells by passive diffusion through the cell membrane.^58^ Therefore, in addition to comparing the delivery capacity of glycoconjugates, our experimental design allows a crude comparison of these three distinct targeting strategies. We evaluated the boron delivery capacity over three time points (5, 30, and 120 min) and a concentration range of 10–400 μM. In more detail, the CAL 27 cells were first incubated with the delivery agents at set concentrations and time points. The cells were then washed and lyzed, and the supernatant from four wells was combined before acidic digestion in order to prepare samples for boron quantification by inductively coupled plasma-mass spectrometry (ICP-MS). The results are summarized in Figure 6. The boron delivery capacity of glycoconjugates **1–3** is superior to that of the clinically employed agents BPA and BSH. While the significant potential embedded in targeting hexose transporter is undeniable, there are a few important findings that emerge when the results are compared to those obtained in our earlier work.^16,20,21^ First, modification of the position 6 seems to be a promising strategy regardless of the stereochemical configuration in the carbohydrate core. Second, the stereochemical configuration does not seem to be as crucial to the functioning principle of the carbohydrate delivery agents as having the appropriate interconnecting atoms in the boron cluster and linker.^21^ Altogether, all three glycoconjugates were found to display similar boron delivery capacities as our previous hit compound **6-oCb-Glc**. Based on the preliminary in vitro assessment reported herein, they should all therefore be viewed as promising delivery agents for BNCT, and future in vivo studies will be necessary to further explore their potential.

## 4 Discussion

The increasing emergence of in-hospital BNCT treatment facilities is having a revolutionizing effect on the entire BNCT field. The last bottleneck of this therapeutic modality are the suboptimal properties displayed by the boron delivery agents in clinical use. Therefore, the development of improved delivery agents is important. In our team, we have focused on the development of carbohydrate-based delivery agents targeting hexose transporters.^16,20,21^ We have previously synthesized and studied a number of glucoconjugates and found delivery agents that outperform the ones in current clinical use in key in vitro preclinical assessment studies. One of the hit molecules was **6-oCb-Glc** in which the position 6 of d-Glc bears a carboranylmethyl substituent. Since the hexose transporters, including the glucose transporters, also recognize other monosaccharides than d-Glc, we here sought to identify which stereochemical configuration in the carbohydrate core would be optimal for the design of the next generation of carbohydrate delivery agents. To this end, we synthesized the corresponding carboranylmethyl derivatives of d-Man, d-All, and d-Gal (epimers of d-Glc) and studied their GLUT affinity, cytotoxicity, and boron delivery capacity as part of a preclinical assessment campaign. All three glycoconjugates were prepared from affordable starting materials via short synthetic routes and carefully characterized by NMR and HRMS. To our surprise, all three delivery agents displayed equally promising biological profiles. They were found to be capable of targeting the glucose transporters in the presence of d-Glc. In addition, they display acceptable in vitro cytotoxicity profiles and, most importantly, significantly boosted boron delivery capacity compared to the delivery agents in current clinical use. While the new glycoconjugates did not outperform the previous hit compound in the in vitro assessment studies, we consider that there is a possibility that more striking differences may yet be uncovered in vivo. In our opinion, the current results are supportive of advancing these molecules to the next stage of the development pipeline.

## Supporting Information Available

NMR spectra and additional details relating to the computational and cellular uptake studies (PDF)
