# Supplementary material for: Sweet Battle of the Epimers—Continued Exploration of Monosaccharide-Derived Delivery Agents for Boron Neutron Capture Therapy
Source: Mol Pharm. 2023 May 3;20(6):3127–39. doi: 10.1021/acs.molpharmaceut.3c00119 (PMC10245378; doi:10.1021/acs.molpharmaceut.3c00119)
Supplement: Supplementary file 1 — mp3c00119_si_001.pdf [file mp3c00119_si_001.pdf]

SUPPORTING INFORMATION

Sweet Battle of the Epimers – Continued Exploration of Monosaccharide-Derived Delivery Agents for Boron Neutron Capture Therapy

Jelena Matović,[1,‡] Katayun Bahrami,[2,‡] Philipp Stockmann,[3] Iris K. Sokka,[1] You Cheng Khng,[1] Mirkka Sarparanta,[1] Evamarie Hey-Hawkins,[3] Jarkko Rautio[2] and Filip S. Ekholm[1*]

[1] Department of Chemistry, University of Helsinki, Finland, P.O. Box 55, FI-00014 Helsinki, Finland

[2] School of Pharmacy, University of Eastern Finland, P.O. Box 1627, FI-70211 Kuopio, Finland

[3] Faculty of Chemistry and Mineralogy, Institute of Inorganic Chemistry, Leipzig University, D-04103 Leipzig, Germany

[‡]Equal contributions

Contact: filip.ekholm@helsinki.fi

Table of contents

1. **NMR Spectra of synthesized compounds** 2
2. **Supplementary information on molecular modeling** 16
3. **Supplementary information on cellular uptake studies** 17

1. NMR Spectra of synthesized compounds

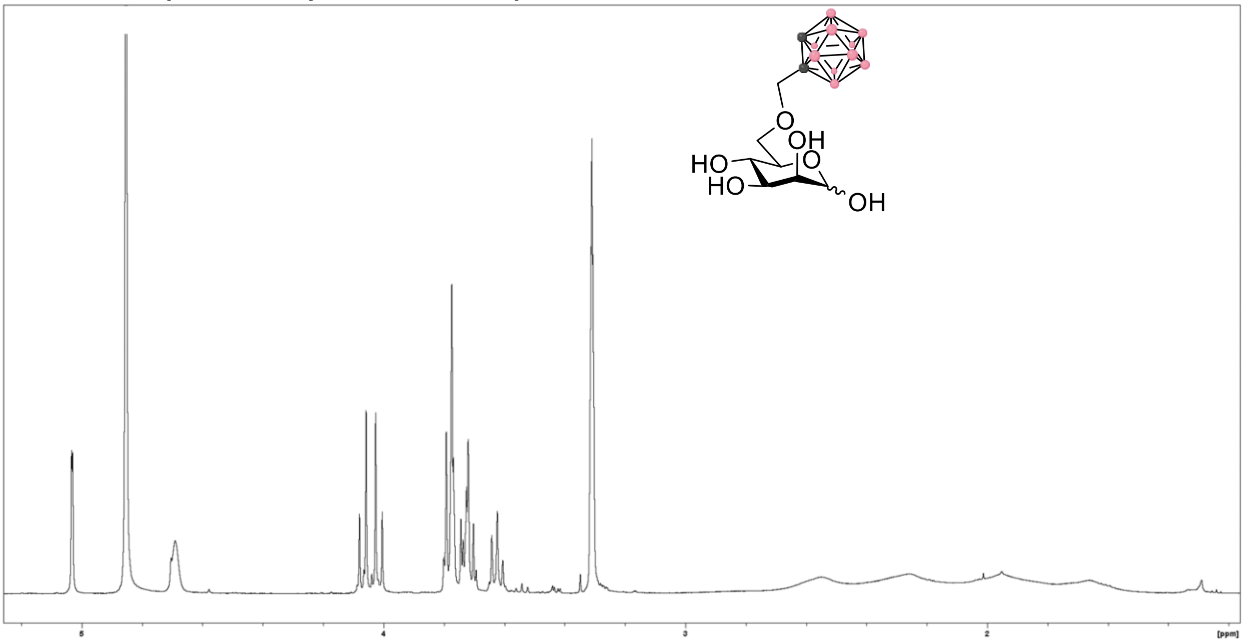

Figure S1. ^{1}H NMR spectrum of **1** (499.83 MHz, 25 °C, CD_3OD).

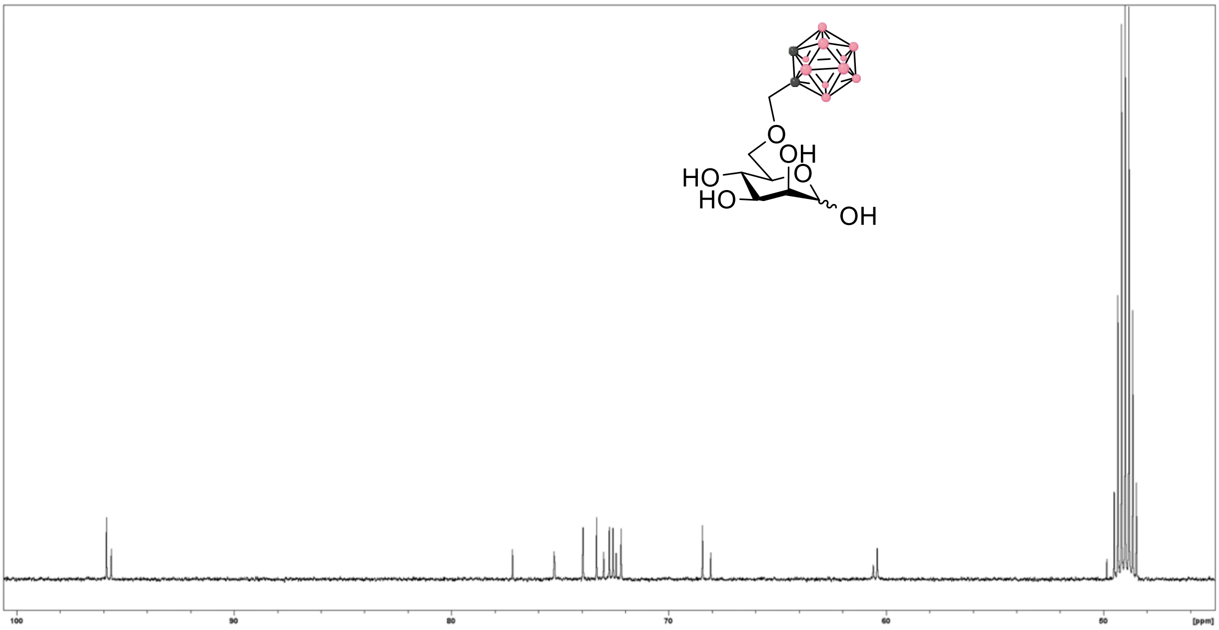

Figure S2. $^{13}C\{^{1}H\}$ NMR spectrum of **1** (125.69 MHz, 25 °C, CD_3OD).

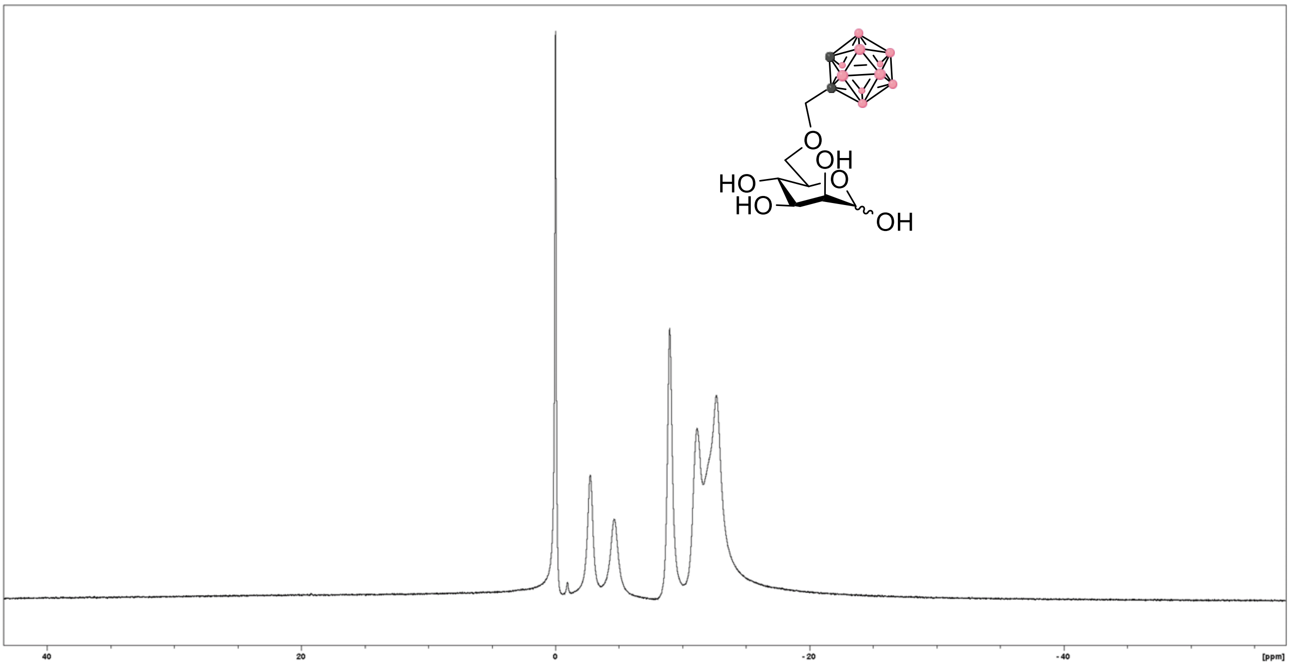

Figure S3. $^{11}B\{^{1}H\}$ NMR spectrum of **1** (160.36 MHz, 25 °C, CD_3OD).

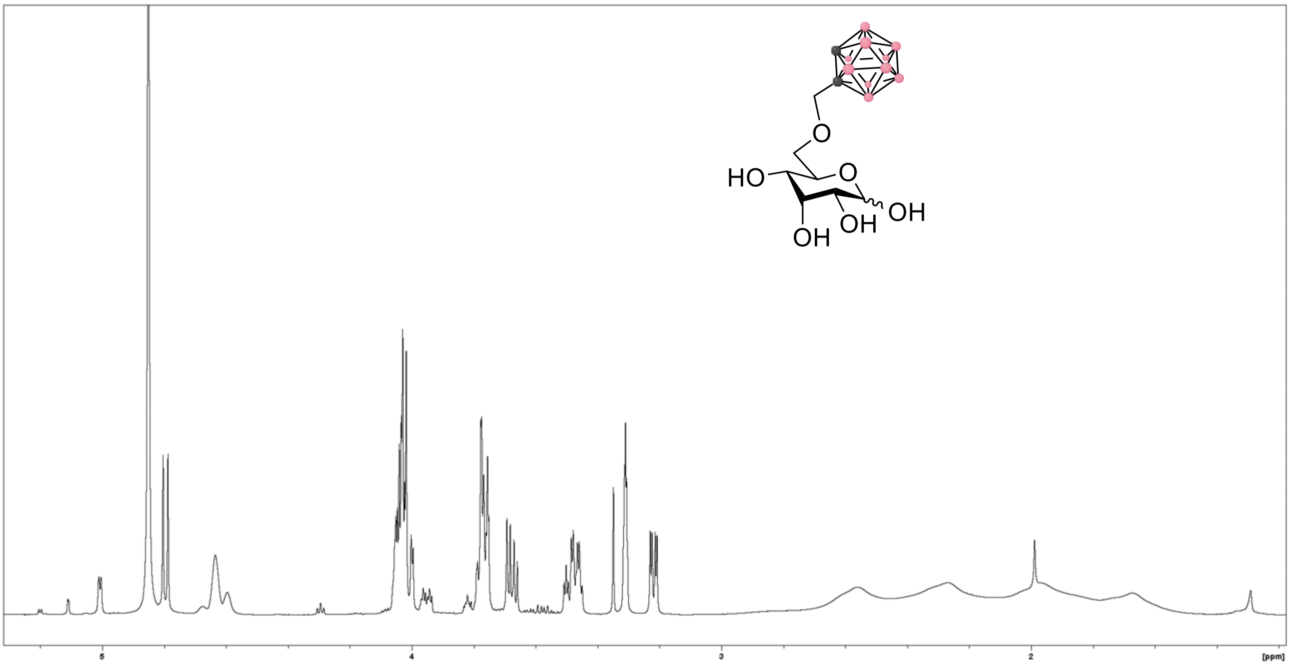

Figure S4. ^{1}H NMR spectrum of **2** (499.83 MHz, 25 °C, CD_3OD).

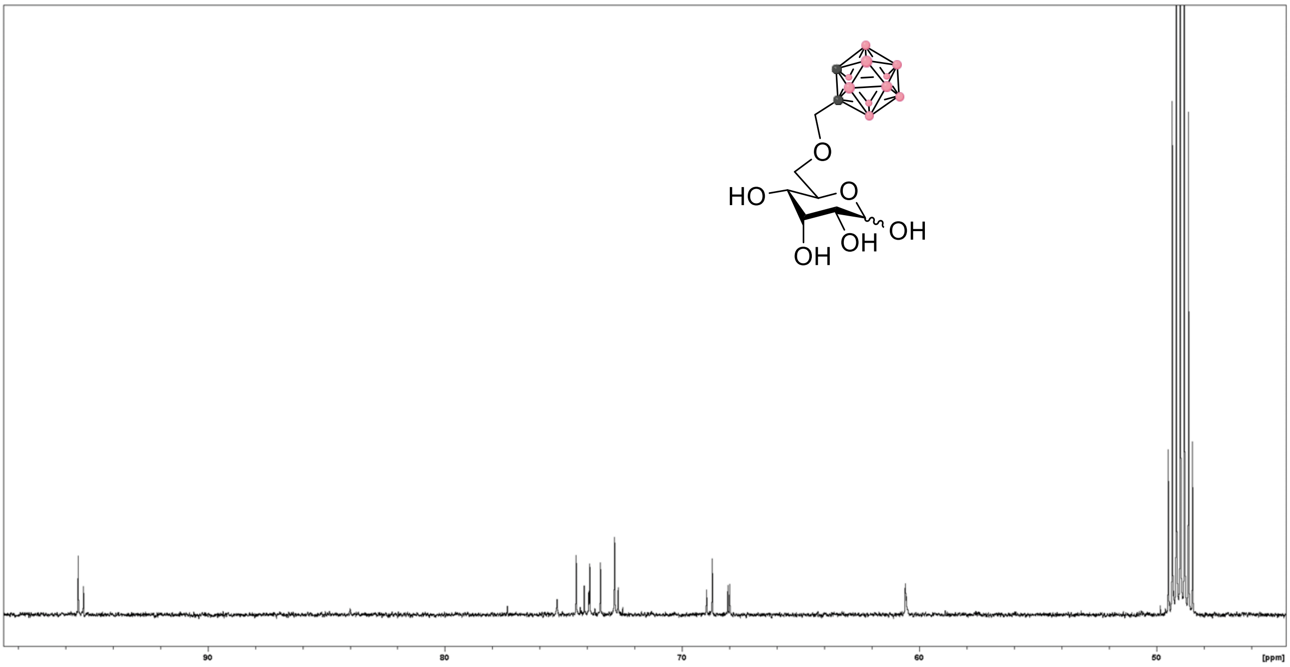

Figure S5. $^{13}C\{^1H\}$ NMR spectrum of **2** (125.69 MHz, 25 °C, CD_3OD).

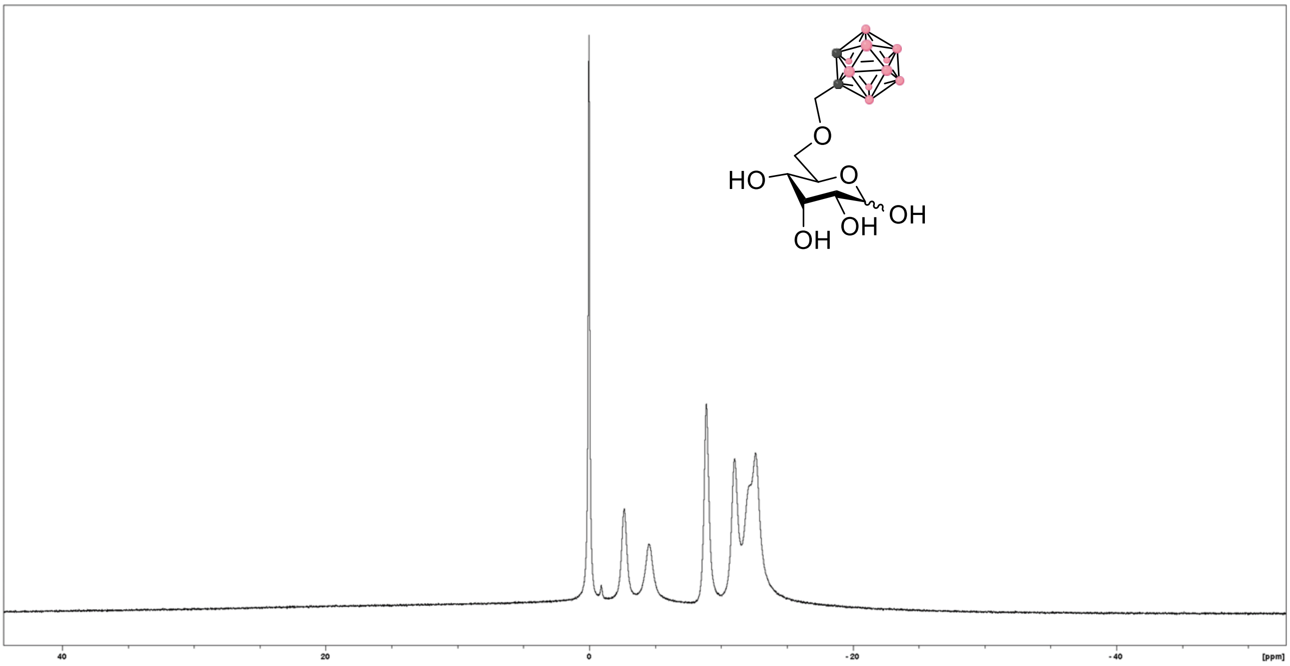

Figure S6. $^{11}B\{^1H\}$ NMR spectrum of **2** (160.36 MHz, 25 °C, CD_3OD).

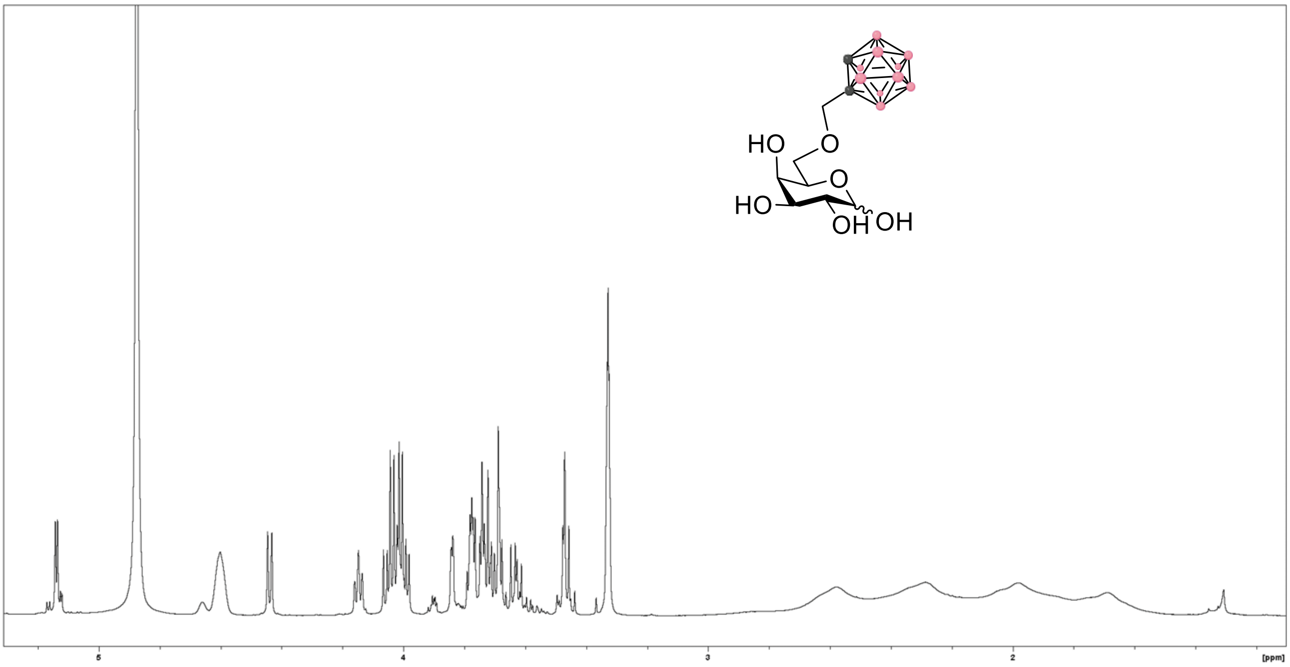

Figure S7. ^{1}H NMR spectrum of **3** (499.83 MHz, 25 °C, CD_3OD).

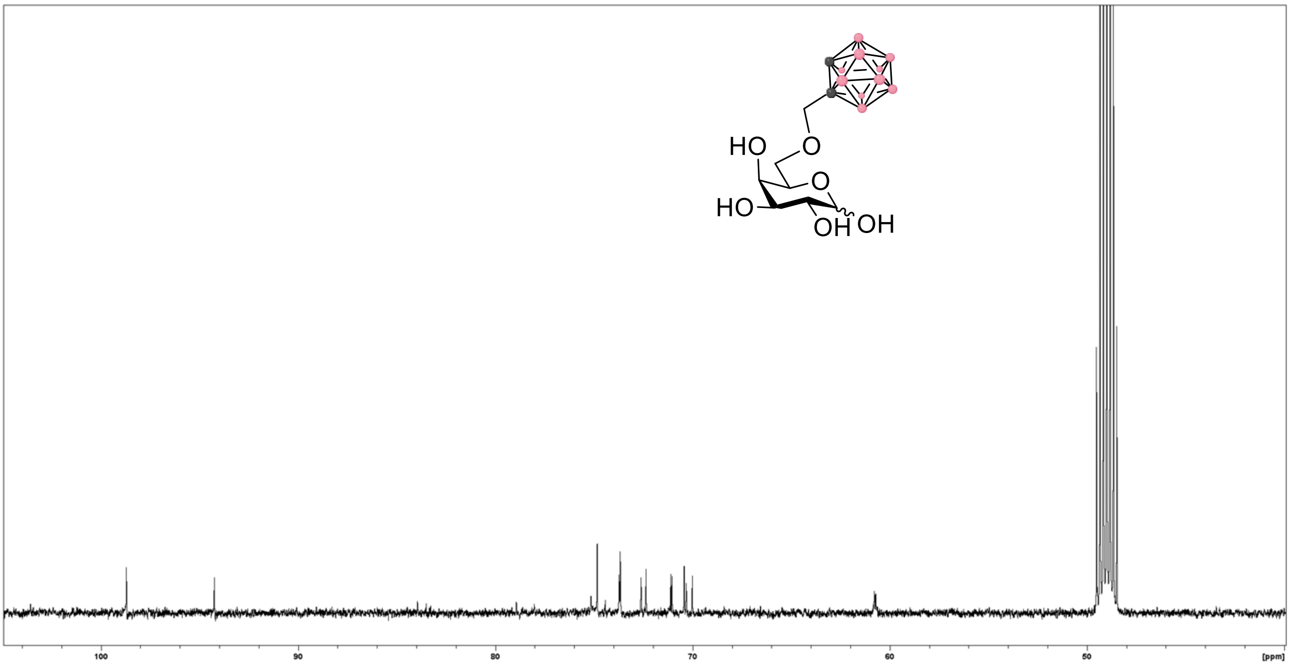

Figure S8. $^{13}C\{^{1}H\}$ NMR spectrum of **3** (125.69 MHz, 25 °C, CD_3OD).

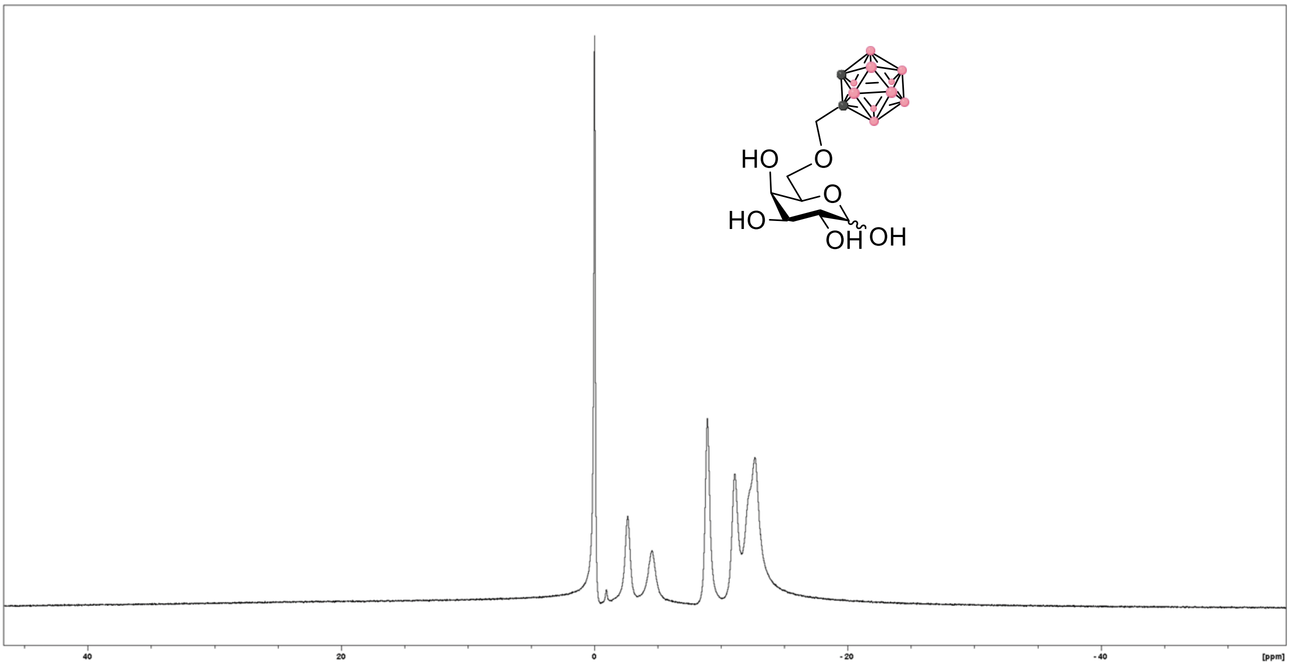

Figure S9. $^{11}B\{^{1}H\}$ NMR spectrum of **3** (160.36 MHz, 25 °C, CD_3OD).

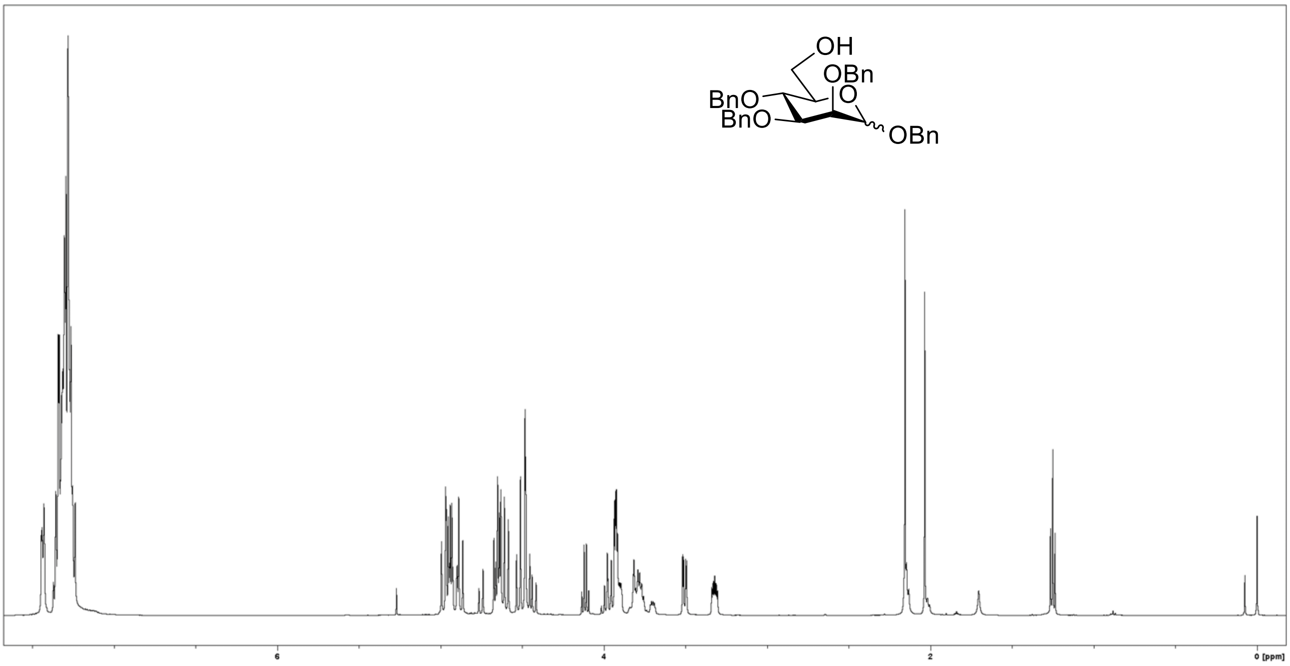

Figure S10. ^{1}H NMR spectrum of **4** (499.83 MHz, 25 °C, $CDCl_3$).

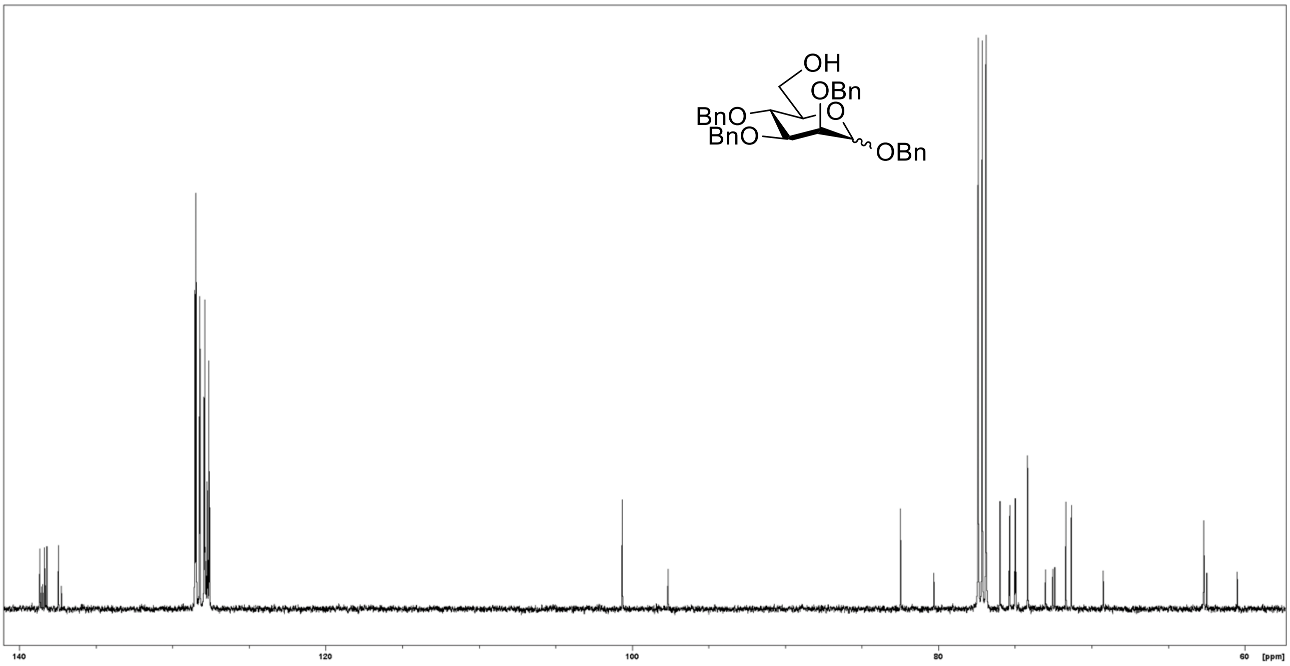

Figure S11. $^{13}C\{^1H\}$ NMR spectrum of **4** (125.69 MHz, 25 °C, $CDCl_3$).

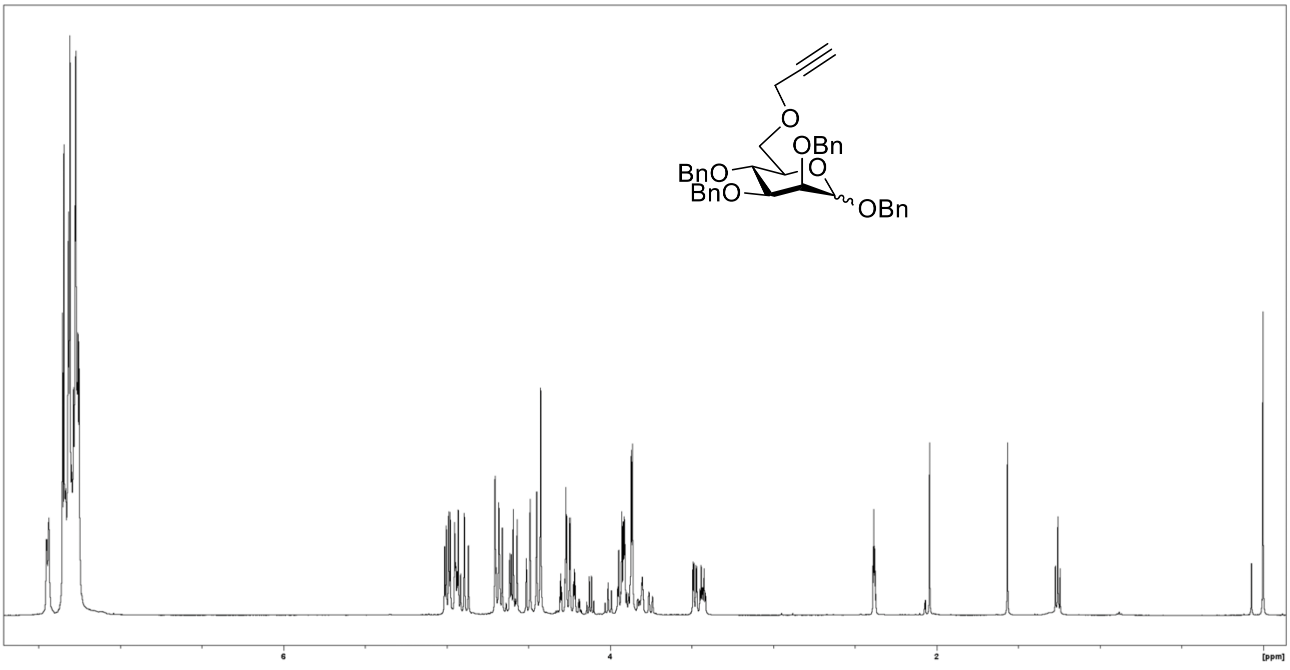

Figure S12. ^1H NMR spectrum of **5** (499.83 MHz, 25 °C, $CDCl_3$).

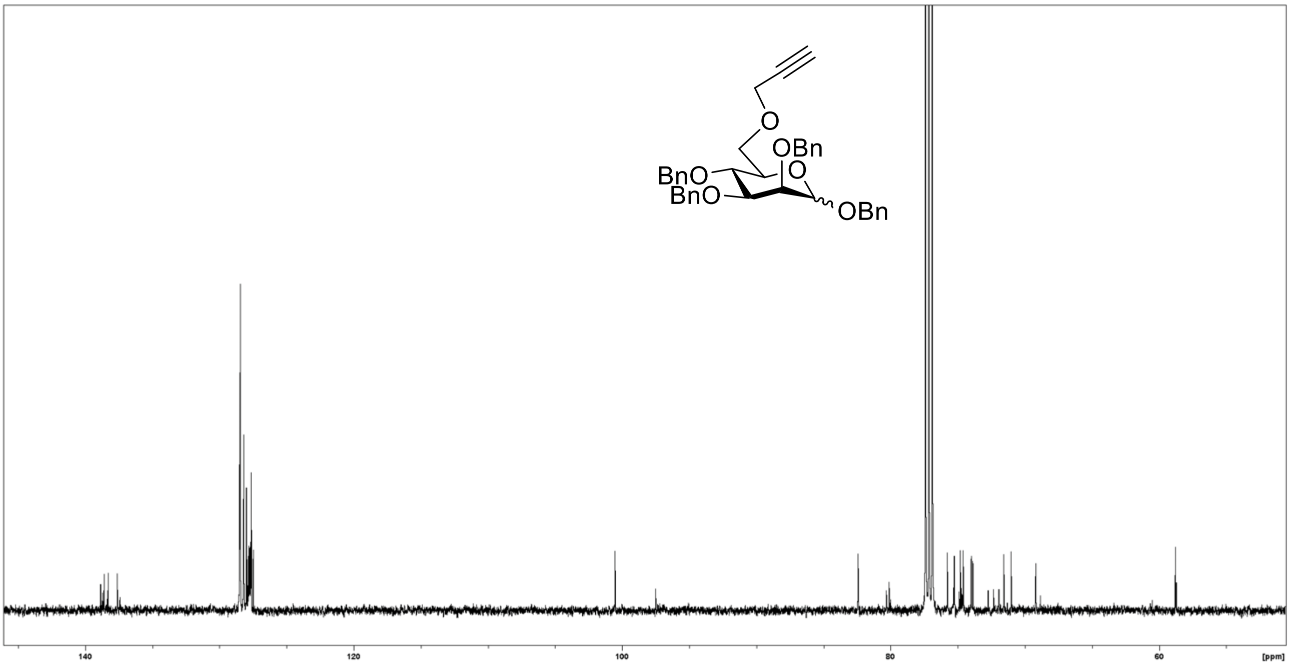

Figure S13. $^{13}C\{^1H\}$ NMR spectrum of **5** (125.69 MHz, 25 °C, $CDCl_3$).

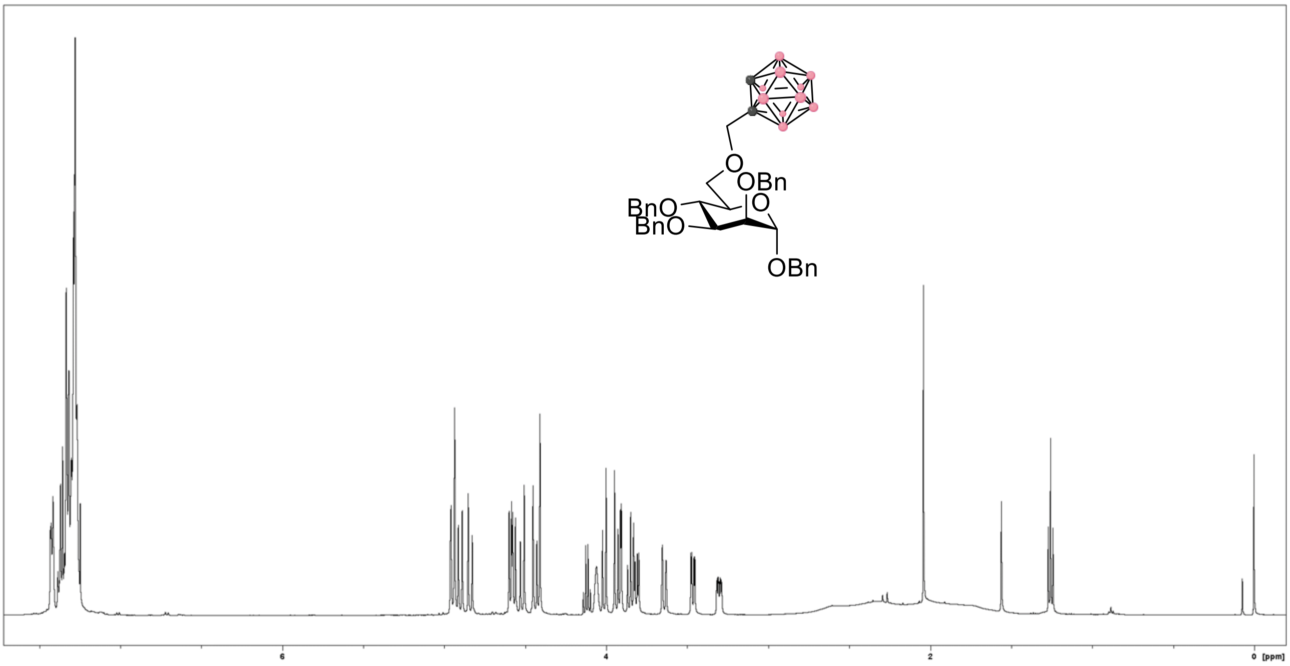

Figure S14. ^1H NMR spectrum of **6** (499.83 MHz, 25 °C, $CDCl_3$).

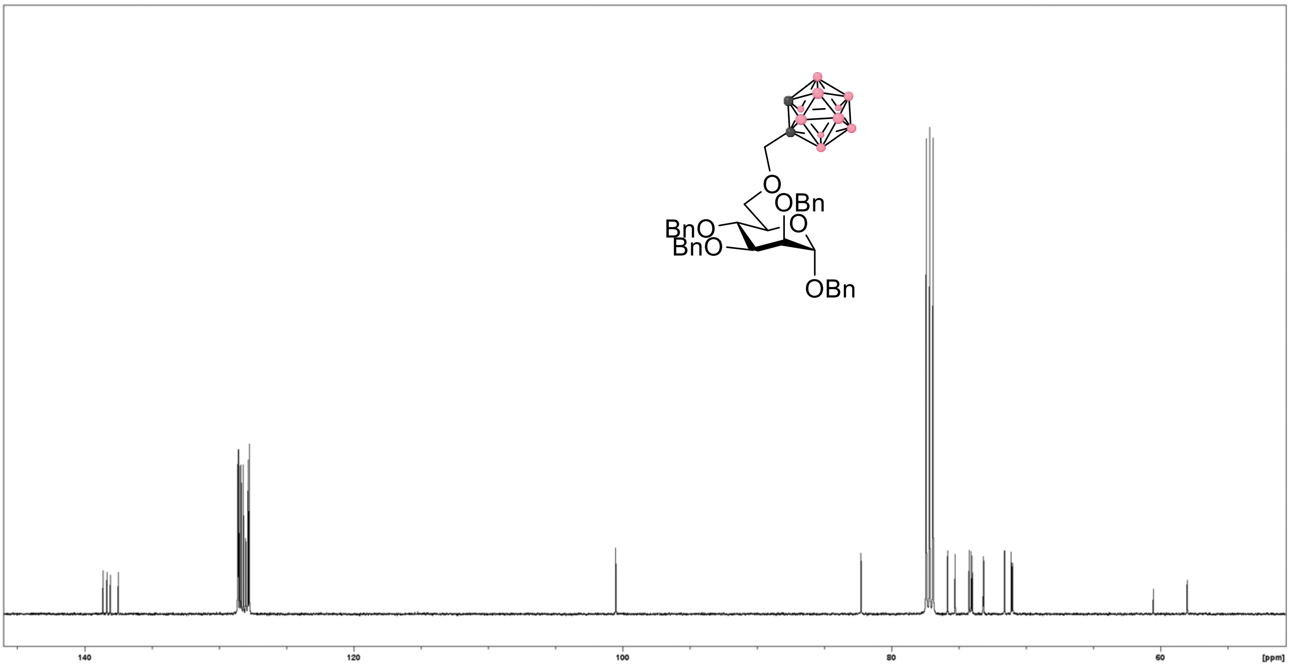

Figure S15. $^{13}C\{^{1}H\}$ NMR spectrum of **6** (125.69 MHz, 25 °C, $CDCl_3$).

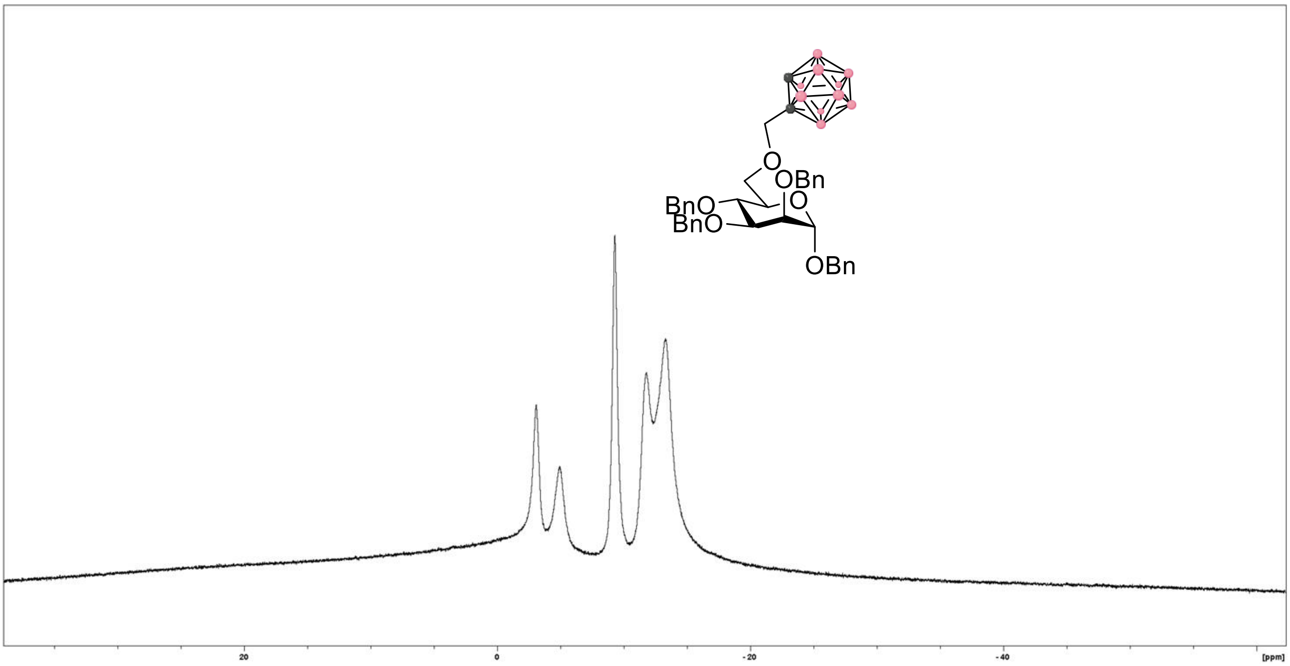

Figure S16. $^{11}B\{^{1}H\}$ NMR spectrum of **6** (160.36 MHz, 25 °C, $CDCl_3$).

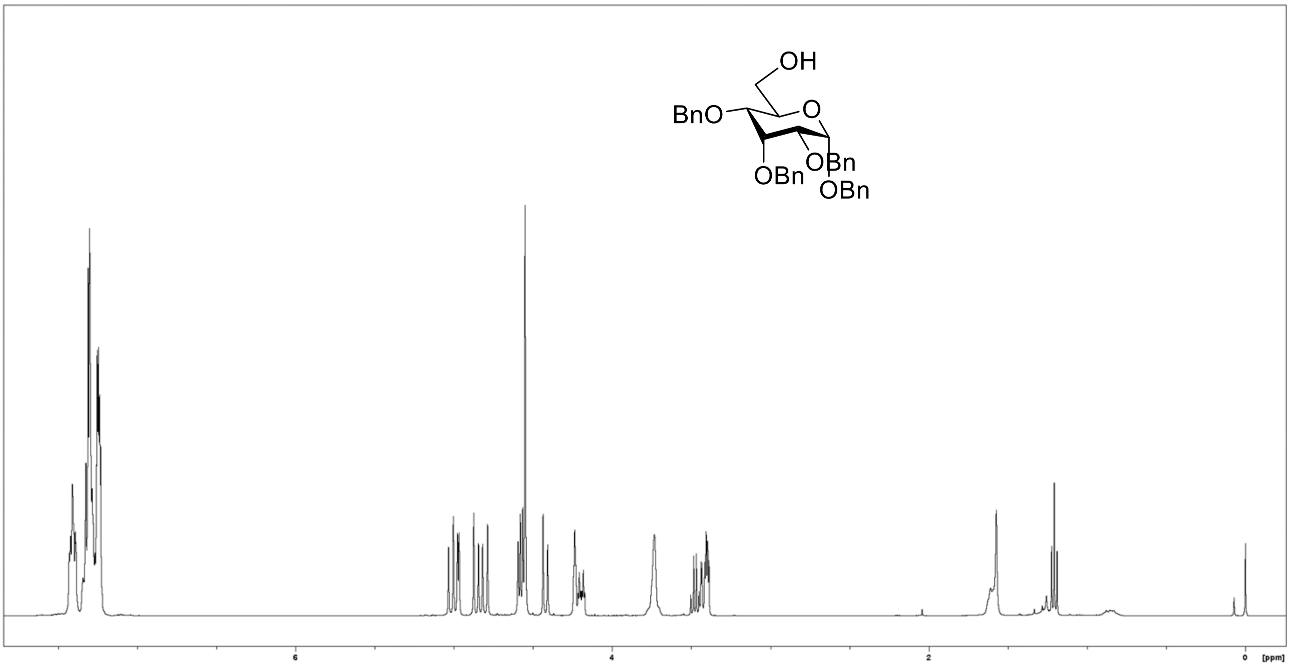

Figure S17. ^{1}H NMR spectrum of **7** (499.83 MHz, 25 °C, $CDCl_3$).

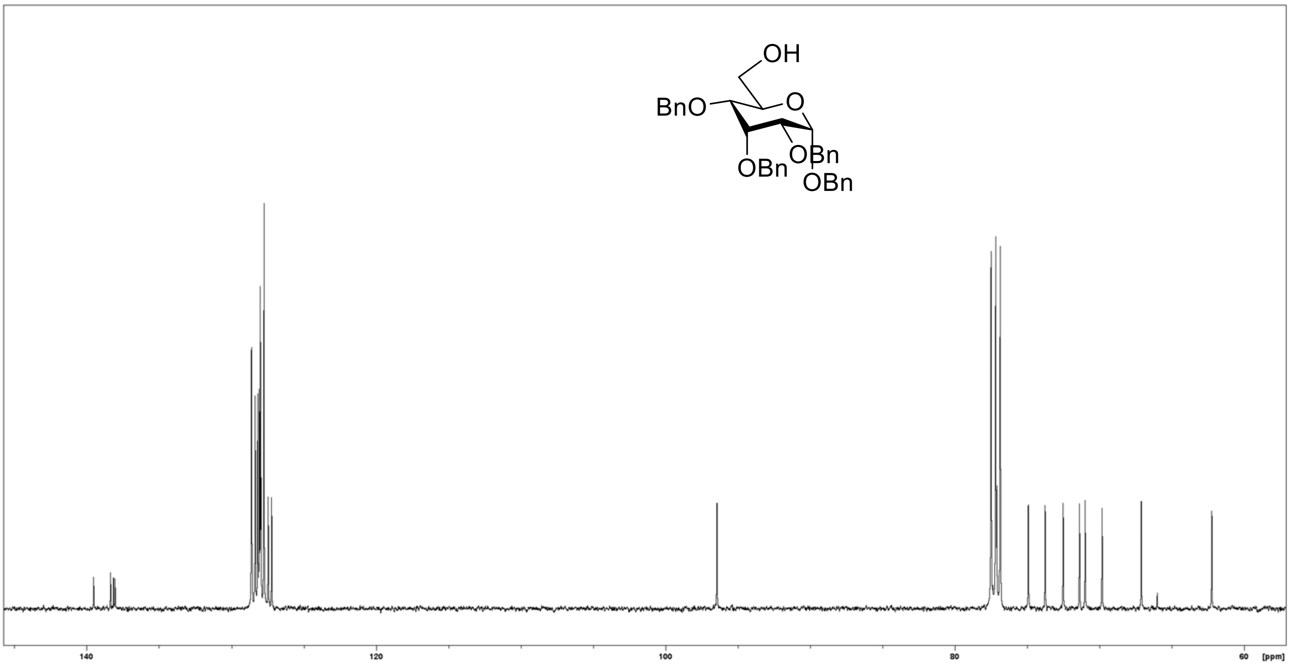

Figure S18. $^{13}C\{^{1}H\}$ NMR spectrum of **7** (125.69 MHz, 25 °C, $CDCl_3$).

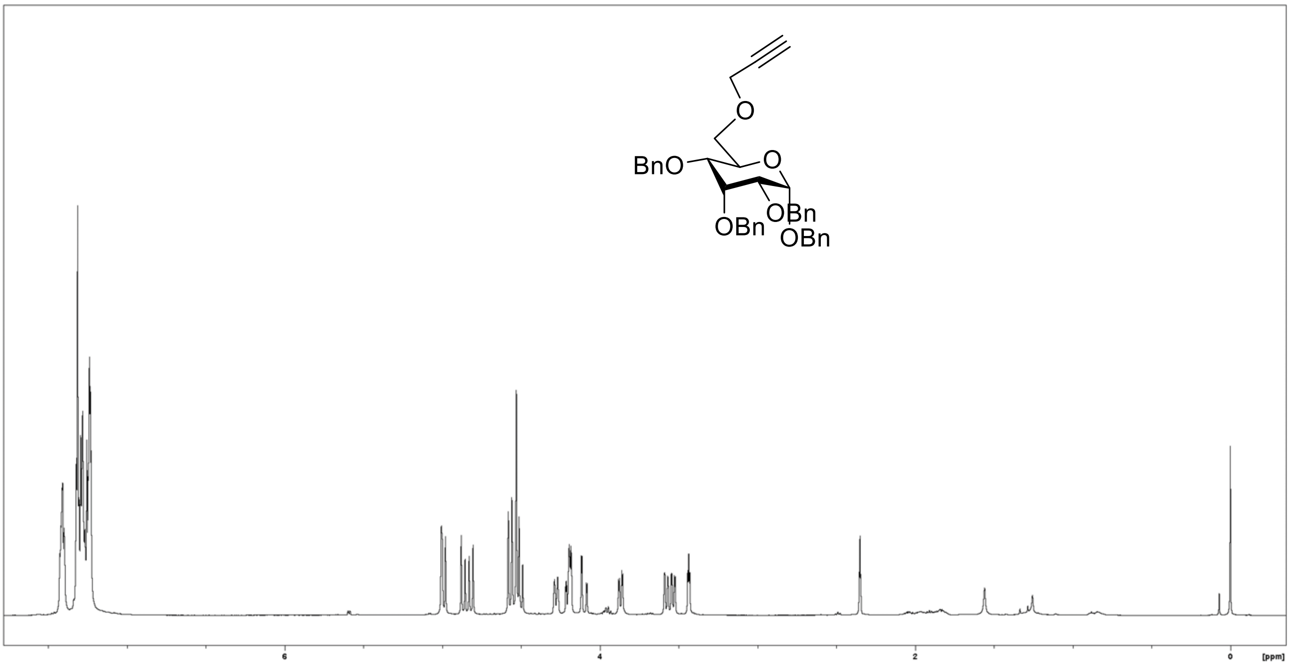

Figure S19. ^{1}H NMR spectrum of **8** (499.83 MHz, 25 °C, $CDCl_3$).

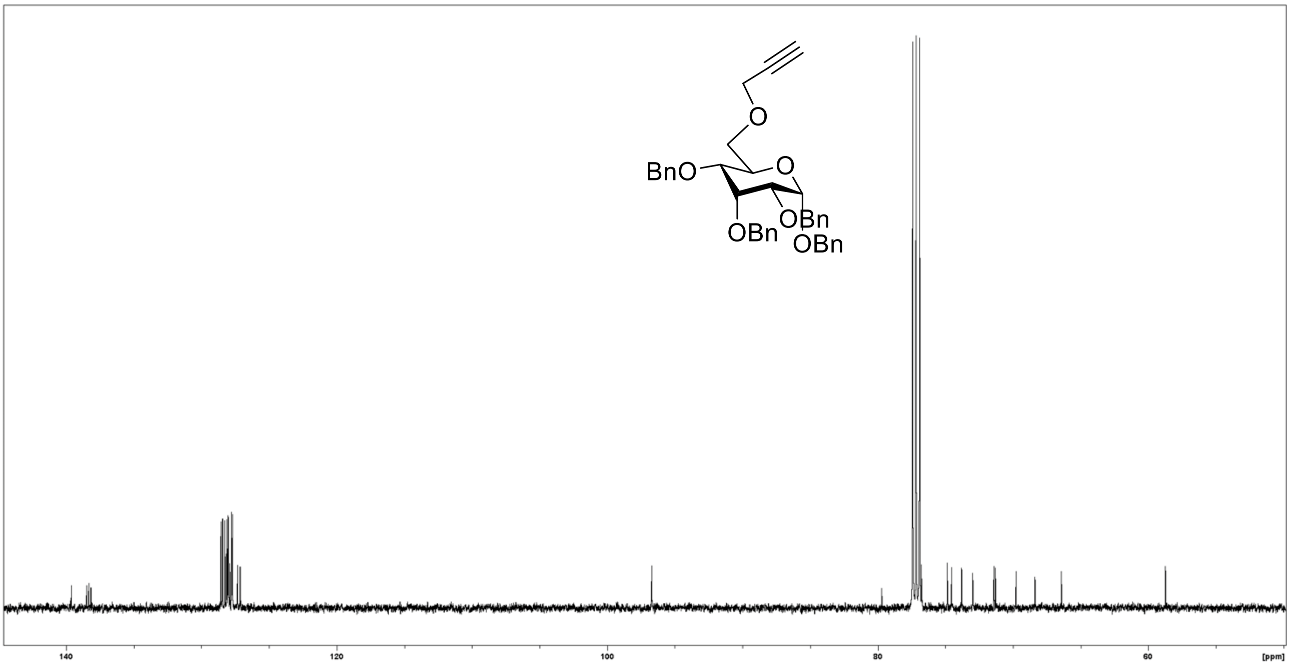

Figure S20. $^{13}C\{^{1}H\}$ NMR spectrum of **8** (125.69 MHz, 25 °C, $CDCl_3$).

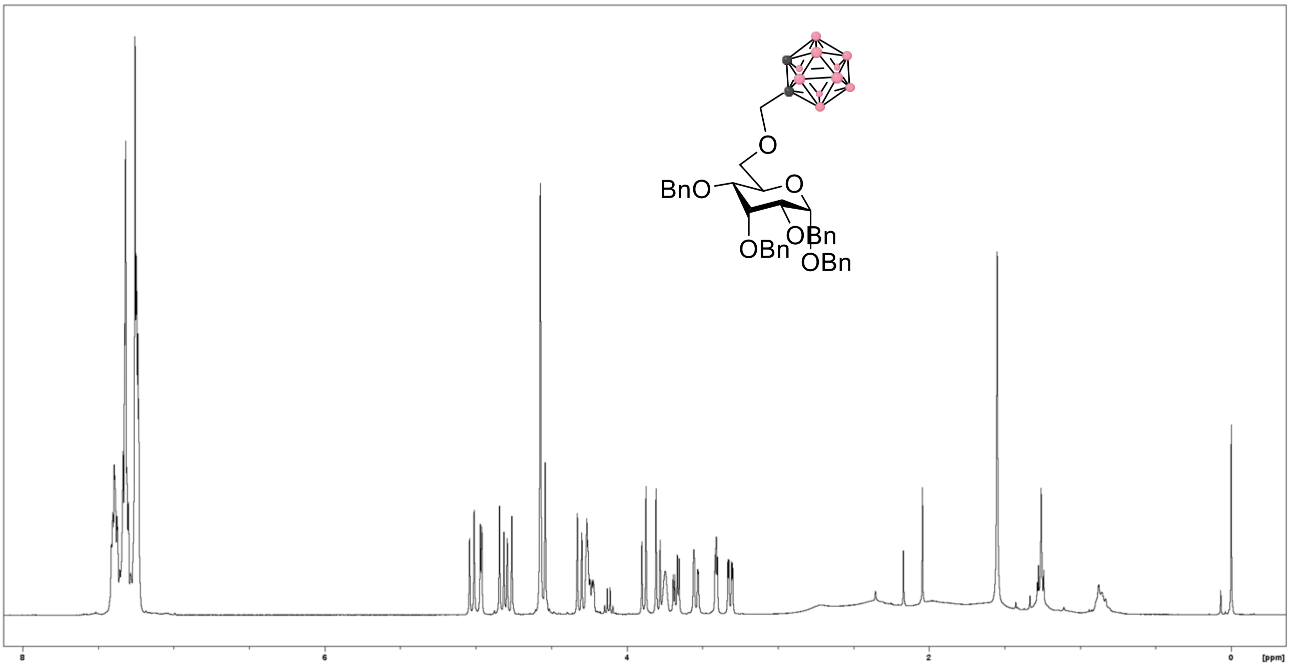

Figure S21. ^{1}H NMR spectrum of **9** (499.83 MHz, 25 °C, $CDCl_3$).

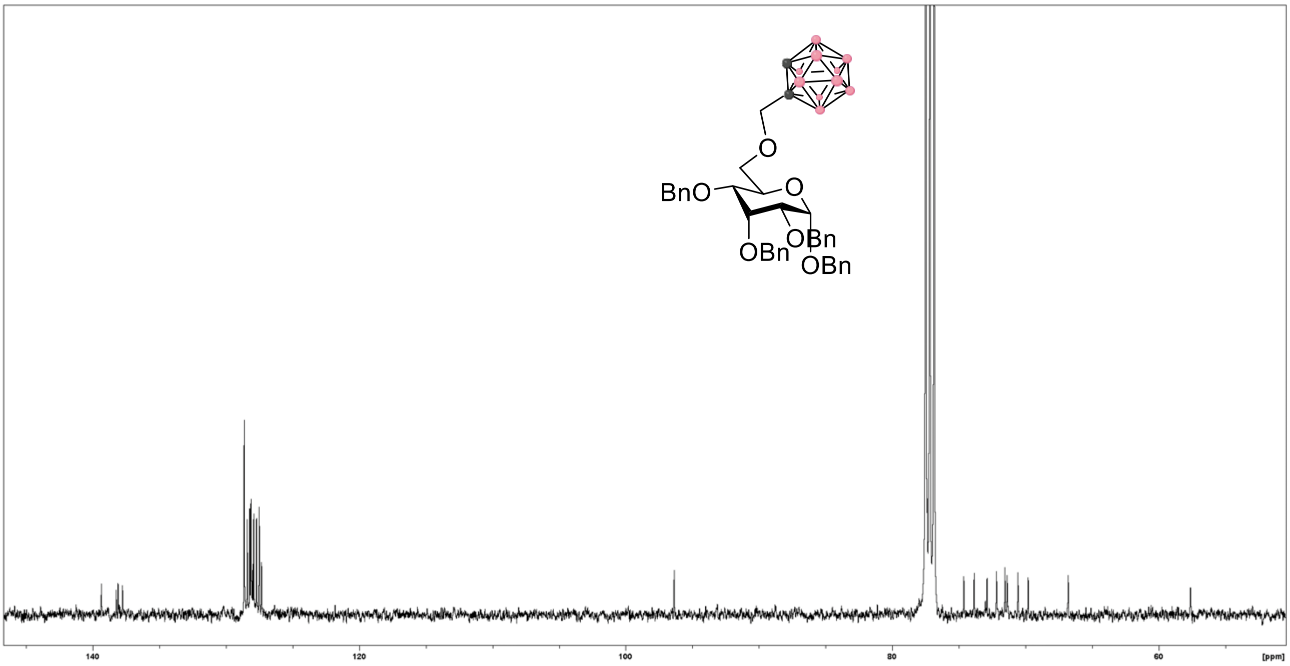

Figure S22. $^{13}C\{^{1}H\}$ NMR spectrum of **9** (125.69 MHz, 25 °C, $CDCl_3$).

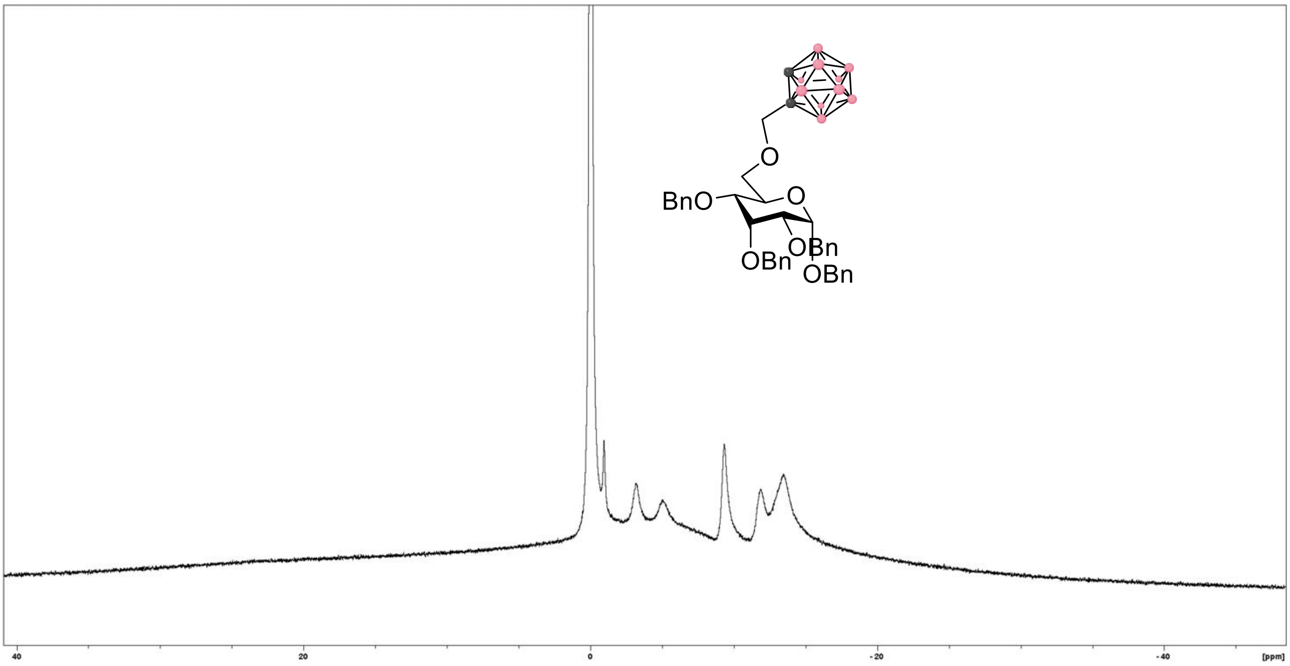

Figure S23. $^{11}B\{^{1}H\}$ NMR spectrum of **9** (160.36 MHz, 25 °C, $CDCl_3$).

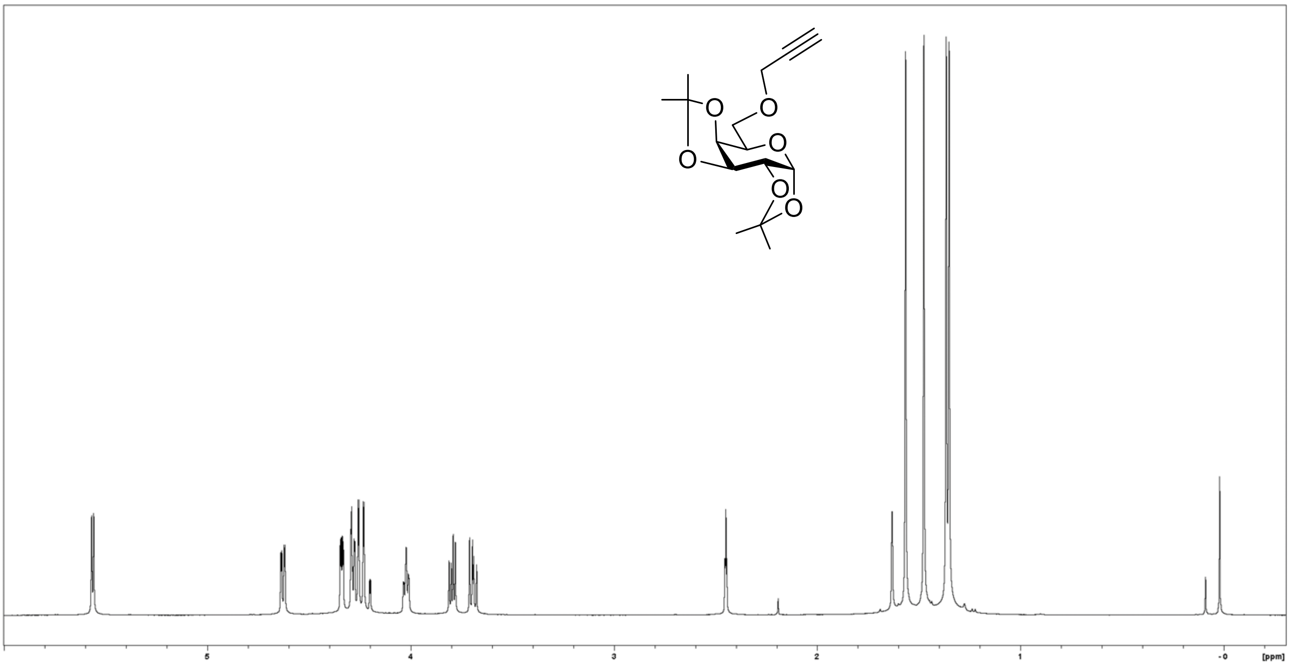

Figure S24. ^{1}H NMR spectrum of **10** (499.83 MHz, 25 °C, $CDCl_3$).

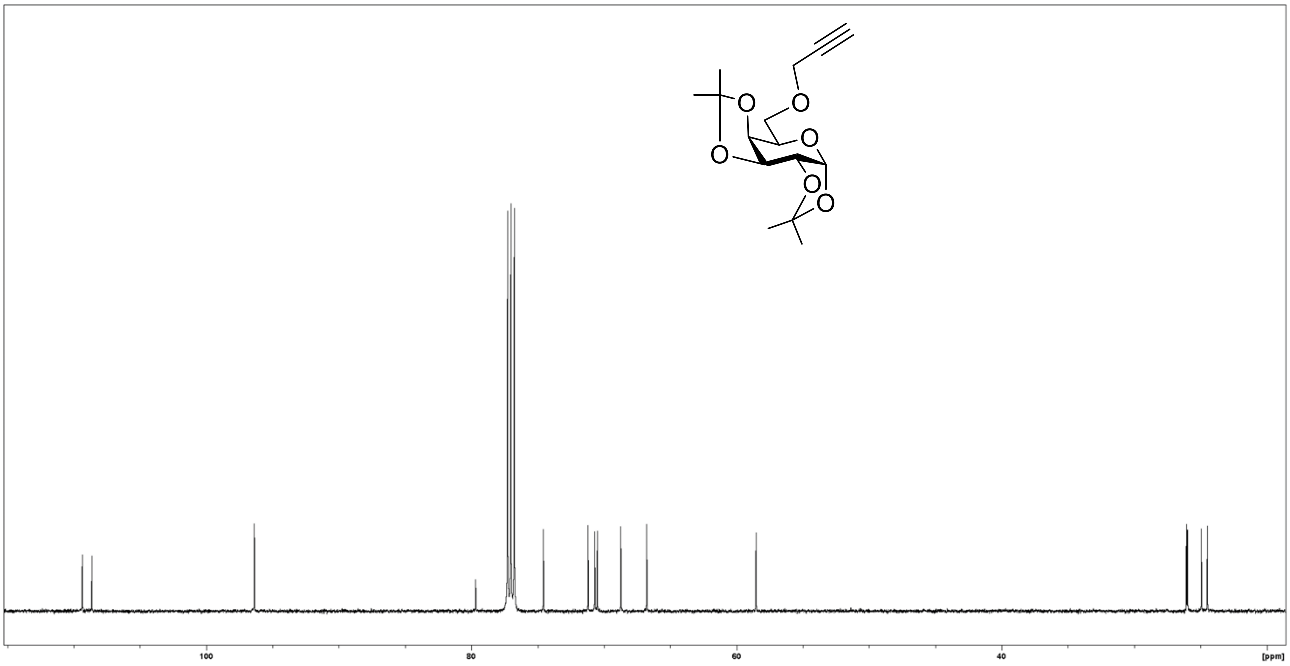

Figure S25. $^{13}C\{^{1}H\}$ NMR spectrum of **10** (125.69 MHz, 25 °C, $CDCl_3$).

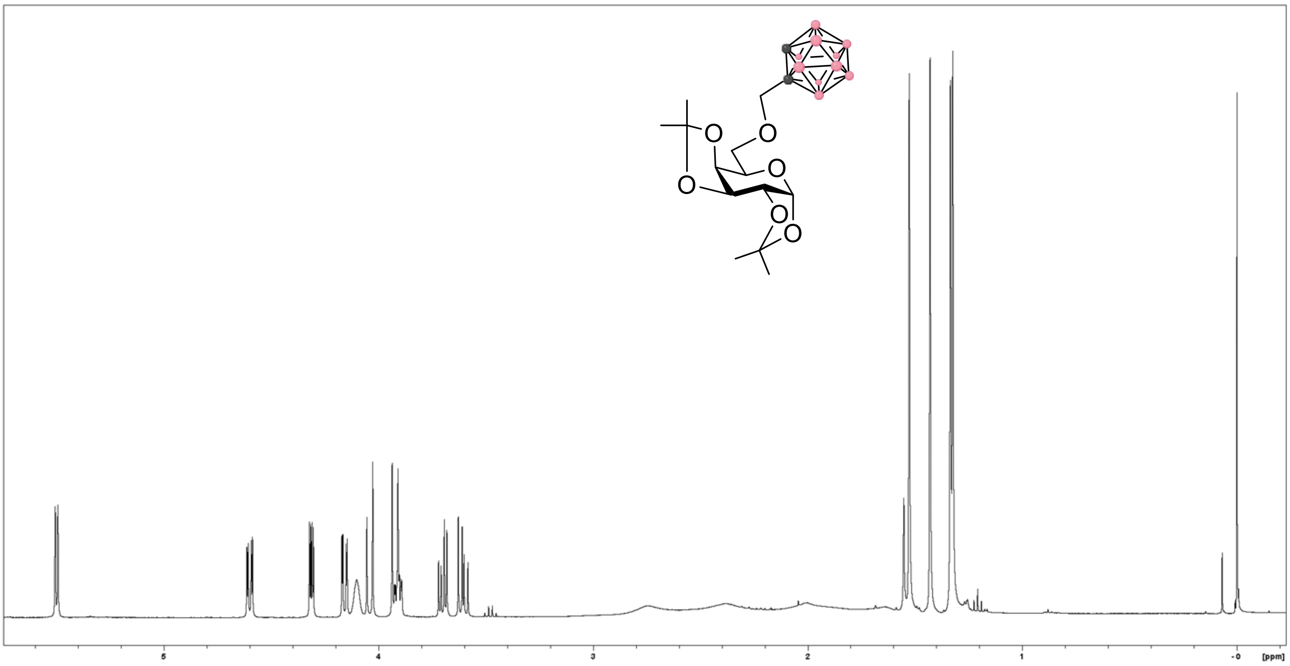

Figure S26. ^{1}H NMR spectrum of **11** (499.83 MHz, 25 °C, $CDCl_3$).

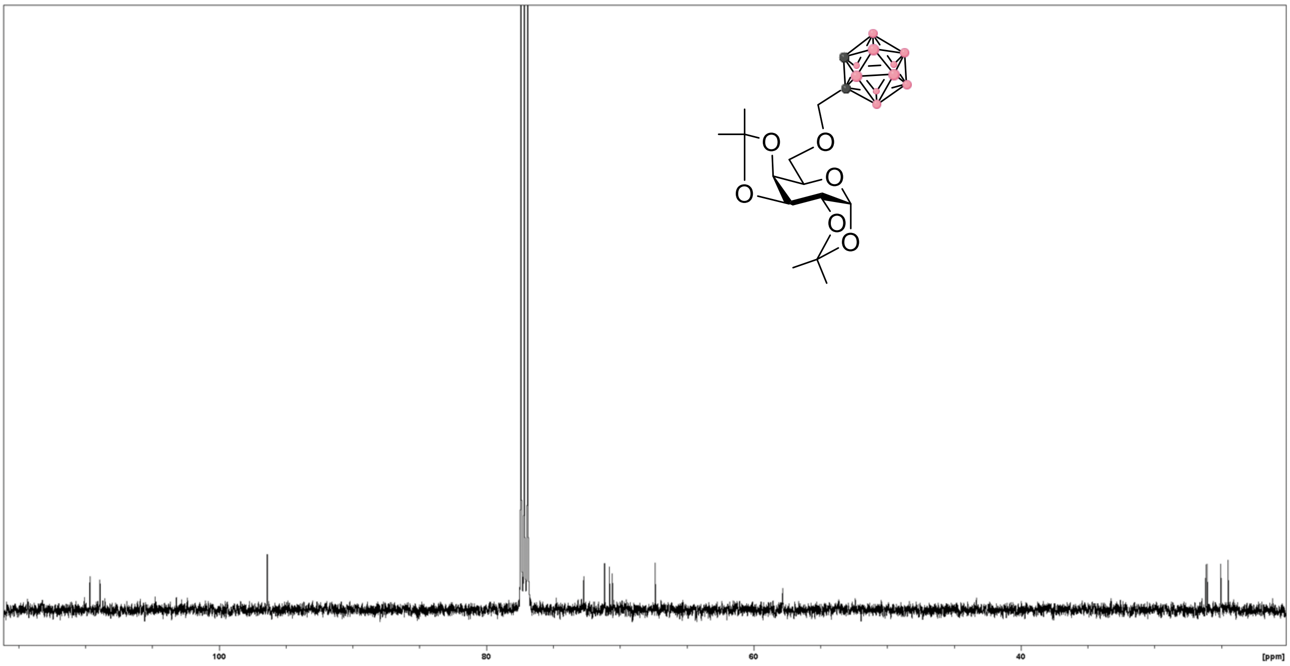

Figure S27. $^{13}C\{^{1}H\}$ NMR spectrum of **11** (125.69 MHz, 25 °C, $CDCl_3$).

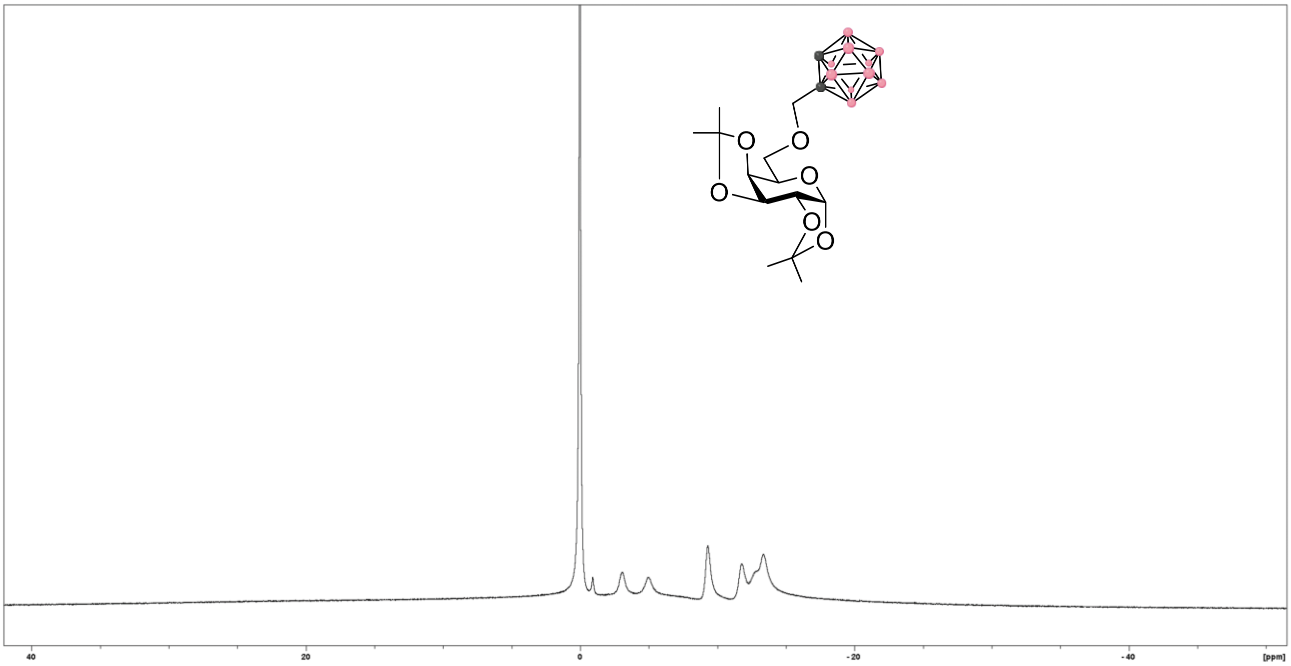

Figure S28. $^{11}B\{^{1}H\}$ NMR spectrum of **11** (160.36 MHz, 25 °C, $CDCl_3$).

2. Supplementary information on molecular modeling

Table S1. Overall mean binding energy (MBE) of each epimer calculated as cluster weighted MBE where the α and β conformations of each ligand has been calculated together, considering the experimentally obtained α:β ratios of both the furanose and pyranose forms.

Outward-open structure	MBE (kcal/mol)	Inward-open structure	MBE (kcal/mol)
**3**	-5.54	**6-oCb-Glc**	-3.9
**6-oCb-Glc**	-5.49	**2**	-3.84
**2**	-5.42	**3**	-3.77
**1**	-5.31	**1**	-3.74
D-glucose	-0.61	D-glucose	-0.59

Table S2. MBE of each modeled structure, that is the α and β anomers for pyranose forms of molecules **1**–**3** and furanose forms of **2** and **3**, calculated as cluster weighted MBE. The ligands are in order from lowest binding energy to highest.

Outward-open structure	MBE (kcal/mol)	Inward-open structure	MBE (kcal/mol)
**3**-pyranose-α	-5.62	**1**-pyranose-β	-4.18
**3**-pyranose-β	-5.62	**3**-pyranose-α	-3.98
**2**-pyranose-α	-5.59	**2**-pyranose-α	-3.94
**1**-pyranose-β	-5.59	**2**-pyranose-β	-3.9
**2**-pyranose-β	-5.47	**3**-pyranose-β	-3.73
**1**-pyranose-α	-5.2	**1**-pyranose-α	-3.58
**3**-furanose-β	-4.98	**3**-furanose-β	-3.37
**3**-furanose-α	-4.96	**2**-furanose-α	-3.27
**2**-furanose-α	-4.65	**3**-furanose-α	-3.16
**2**-furanose-β	-4.27	**2**-furanose-β	-2.85

1. Supplementary information on cellular uptake studies

Table S3. Combined results featuring the Michaelis−Menten kinetic parameters V_{max} and K_m at 5, 30 and 120 min for BPA, BSH and glycoconjugates **1**–**3**. (Vmax is given as µg B/mg protein; Km is given as µM)

Compounds	V_{max} at 5 min	K_m at 5 min	V_{max} at 30 min	K_m at 30 min	V_{max} at 120 min	K_m at 120 min
BPA	1.420 ± 1.038	665.0 ± 688.6	1.282 ± 0.1024	399.8 ± 52.47	2.866 ± 0.4014	801.2 ± 151.9
BSH	0.1824 ± 0.1561	608.2 ± 755.3	1.154 ± 0.4990	2565 ± 1242	0.6392 ± 0.1228	538.1 ± 155.1
**1**	11.13 ± 4.134	680.8 ± 356.0	2.092 ± 0.07266	42.06 ± 5.701	4.741 ± 0.3989	96.67 ± 22.94
**2**	32.20 ± 13.82	1873 ± 933.0	3.819 ± 0.2161	98.33 ± 15.59	15.57 ± 4.914	898.3 ± 374.0
**3**	10.05 ± 0.8103	418.0 ± 54.60	17.79 ± 5.239	1916 ± 653.1	3.638 ± 0.1727	70.43 ± 10.71
